# Pathway Analysis Reveals Common Pro-Survival Mechanisms of Metyrapone and Carbenoxolone after Traumatic Brain Injury

**DOI:** 10.1371/journal.pone.0053230

**Published:** 2013-01-09

**Authors:** Helen L. Hellmich, Daniel R. Rojo, Maria-Adelaide Micci, Stacy L. Sell, Deborah R. Boone, Jeanna M. Crookshanks, Douglas S. DeWitt, Brent E. Masel, Donald S. Prough

**Affiliations:** Department of Anesthesiology, University of Texas Medical Branch, Galveston, Texas, United States of America; Rutgers University, United States of America

## Abstract

Developing new pharmacotherapies for traumatic brain injury (TBI) requires elucidation of the neuroprotective mechanisms of many structurally and functionally diverse compounds. To test our hypothesis that diverse neuroprotective drugs similarly affect common gene targets after TBI, we compared the effects of two drugs, metyrapone (MT) and carbenoxolone (CB), which, though used clinically for noncognitive conditions, improved learning and memory in rats and humans. Although structurally different, both MT and CB inhibit a common molecular target, 11β hydroxysteroid dehydrogenase type 1, which converts inactive cortisone to cortisol, thereby effectively reducing glucocorticoid levels. We examined injury-induced signaling pathways to determine how the effects of these two compounds correlate with pro-survival effects in surviving neurons of the injured rat hippocampus. We found that treatment of TBI rats with MT or CB acutely induced in hippocampal neurons transcriptional profiles that were remarkably similar (i.e., a coordinated attenuation of gene expression across multiple injury-induced cell signaling networks). We also found, to a lesser extent, a coordinated increase in cell survival signals. Analysis of injury-induced gene expression altered by MT and CB provided additional insight into the protective effects of each. Both drugs attenuated expression of genes in the apoptosis, death receptor and stress signaling pathways, as well as multiple genes in the oxidative phosphorylation pathway such as subunits of NADH dehydrogenase (Complex1), cytochrome c oxidase (Complex IV) and ATP synthase (Complex V). This suggests an overall inhibition of mitochondrial function. Complex 1 is the primary source of reactive oxygen species in the mitochondrial oxidative phosphorylation pathway, thus linking the protective effects of these drugs to a reduction in oxidative stress. The net effect of the drug-induced transcriptional changes observed here indicates that suppressing expression of potentially harmful genes, and also, surprisingly, reduced expression of pro-survival genes may be a hallmark of neuroprotective therapeutic effects.

## Introduction

To date, all pharmacotherapeutic agents in clinical trials for treatment of traumatic brain injury (TBI) have failed to show efficacy, suggesting a need for more effective pre-clinical screening of novel therapeutic compounds [1]. Significant factors that contribute to lifelong disability after TBI are learning and memory deficits associated with damage to the hippocampus. Thus, our screening efforts focused on compounds that provide neuroprotection in the hippocampus [2]. Although the large group of compounds that have been shown to reduce neuronal damage in animal models of hippocampal injury are structurally and functionally diverse and target multiple cell signaling pathways [3], their common neuroprotective effects suggest a common mechanism of action. Elucidating the common pro-survival mechanisms shared by these drugs could provide selective criteria to choose compounds as potential treatments for TBI. Rather than a reductionist gene-by-gene strategy, recent advances in systems biology allow us to interrogate disease-relevant networks on a genome-wide scale [4], [5]. Systematically comparing common changes in cell signaling pathways generated by otherwise diverse neuroprotective compounds could provide useful mechanistic insights into the essential elements of neuroprotection.

Ideally, in order to counteract hippocampal dysfunction, an effective therapeutic agent would have both neuroprotective and nootropic (memory and cognitive enhancing) properties. In order to focus on the drug-induced alterations in known cell signaling pathways, as opposed to the functional consequences of drug treatment, we investigated two compounds, metyrapone (MT) and carbenoxolone (CB), that possess neuroprotective and nootropic properties but have been used clinically for nonneurologic indications. Metyrapone is used to test for adrenal insufficiency [6] and CB—a derivative of 18-glycyrrhetinic acid and a mineralocorticoid agonist—has been used for a variety of purposes, including treatment of peptic ulcers [7]. Both exert their neuroprotective and memory enhancing effects, in part, by inhibition of a common molecular target. Each compound inhibits the gene coding for 11β hydroxysteroid dehydrogenase type 1 (11βHSD1), which converts inactive cortisone to active cortisol in the brain [8], [9]. Both also act through additional unidentified cellular signals [10], [11]. Chronically elevated cortisol levels are traditionally associated with both hippocampal atrophy and hippocampal-dependent learning and memory deficits in aging humans [12]. Thus, both the effects of MT on improving memory consolidation and retrieval in rats [13], [14], and CB on improving verbal fluency and memory in normal and diabetic elderly men [9] imply that these effects are mediated through inhibition of 11βHSD1 [12].

Additionally, glucocorticoid-mediated oxidative damage in the rat hippocampus has been associated with cognitive deficits [15]. Other studies have also shown that injury- and stress-induced release of glucocorticoids increases glutamate release in the prefrontal cortex and hippocampus. Through stimulation of endocannabinoids, glucocorticoids also influence GABAergic, noradrenergic, cholinergic and serotonergic neurotransmission in the brain [16]. However, other reported pro-survival effects of CB and MT appear to be mediated by divergent mechanisms. For example, CB is often associated with induction of heat shock proteins and non-gap junction mediated effects on synaptic transmission [17]–[19]. Similarly, MT is found to influence sleep, depression, cytochrome P450 genes, and cyclic AMP response element binding protein [8], [20]–[23]. These effects provide evidence that these two drugs have multiple mechanisms of action associated with their neuroprotective and nootropic effects.

Although both MT and CB have undesirable side-effects that preclude their long-term clinical use [24]–[26], their beneficial effects may still be useful as short-term treatments acutely after TBI. In the present study, these drugs served as a training set to test our hypothesis that neuroprotective drugs that reduce neuronal damage in the hippocampus would alter TBI-induced gene expression in an identifiable pattern of cell signaling pathways associated with cell survival or cell death. We addressed this hypothesis using gene expression analysis of laser-captured pure populations of surviving hippocampal CA3 neurons. We used Ingenuity pathway analysis to examine changes in injury-induced cell signaling pathways associated with MT and CB treatment. We then compared these data to similar data obtained from separate pure populations of dying or degenerating neurons [27]. Because previous studies showing neuroprotection with MT have used a pre-treatment regimen [10], and we wanted to produce similar protective effects, we administered both drugs before TBI and estimated neuronal damage by counting degenerating, Fluoro-Jade-positive hippocampal neurons 24 hours after TBI.

We found that the protective effects of MT and CB in the injured rat hippocampus were associated with similar patterns of changes in injury-induced gene expression, i.e., reduced oxidative stress response, suppressed expression of potentially harmful genes and, surprisingly, reduced expression of pro-survival genes.

## Materials and Methods

### Animals

Adult male Sprague-Dawley rats (400–500 g) from Charles River (Portland, Maine) were housed two per cage with food and water *ad libitum* in a *vivarium* with these constant conditions: light cycle (6:00–18:00) temperature (21°C–23°C), and humidity (40%–50%). All animal experiments were approved by the Institutional Animal Care and Use Committee of the University of Texas Medical Branch, Galveston, Texas, and conducted according to the National Institutes of Health Guide for the Care and Use of Laboratory Animals (8^th^ Edition, National Research Council).

### Experimental Design

Rats were randomly assigned to one of four treatment groups (n = 6 per group). All treatments were administered 30 min prior to moderate TBI: 1) sham injury plus saline (0.1 ml/kg sc; SHAM); 2) moderate TBI plus saline (0.1 ml/kg sc; TBI); 3) moderate TBI plus metyrapone (100 mg/kg; TBI+MT); 4) moderate TBI plus carbenoxolone (30 mg/kg; TBI+CB). Brains were collected at 4 and 24 h post surgery, immediately frozen on dry ice and stored at −80°C for later Fluoro-Jade (FJ) staining and laser capture microdissection (LCM) for gene expression analysis.

### Fluid percussion traumatic brain injury

Moderate fluid percussion TBI was performed as previously described [28], [29]. Rats were anesthetized with 4% isoflurane, intubated, and mechanically ventilated (NEMI Scientific; New England Medical Instruments, Medway, MA) with 1.5–2.0% isoflurane in oxygen∶air (70∶30), and received either moderate (1.8–2.0 atm) fluid-percussion TBI or sham injury (surgical preparation without TBI). Rectal temperature was monitored using a Physitemp Thermalert Model TH-8 (Physitemp Instruments, Inc., Clifton, NJ), and maintained at 37°C throughout the procedure, using a thermostatically controlled water blanket (Gaymar Industries, Inc., Orchard Park, NY).

### Fluoro-Jade (FJ) staining

Brains were embedded in OCT and 10 µm sections were stained with 0.001% FJ and counterstained with 1% cresyl violet. FJ-positive neurons in the CA1–CA3 hippocampal subfields were counted as previously described [30].

### Laser Capture Microdissection

Using a PixCell IIe laser capture microscope with an infrared diode laser (MDS Analytical Technologies, Sunnyvale, CA), we located surviving, FJ-negative neurons that were adjacent to dying, FJ-positive neurons as described in Rojo *et al.* [27]. We obtained pools of surviving, FJ-negative neurons from the CA3 region of the hippocampus for microarray analysis. To minimize contamination from adjacent dying neurons, surviving neurons from the ipsilateral hippocampus (directly under the injury site) were captured using the smallest laser spot size (7.5 micron) with a power setting of 75–100 mW and pulse duration of 0.45–0.85 ms. These last two settings were adjusted, as necessary, for optimal cell capture. Hippocampal neurons are estimated to be 20–30 µm in diameter, thus we calculated that 3 captures would yield the total RNA equivalent of one whole neuron, so 200 cell equivalents represent a total of 600 captures. Approximately 200 FJ-negative pyramidal neurons were collected from the CA3 region of each rat and a total of approximately 600 neurons were pooled for each of two biological replicates (n = 3 rats per replicate).

### Microarray Analysis

Total RNA was prepared using the RNAqueous Micro kit (Ambion), and the RNA samples were sent to GenUs BioSystems (Northbrook, IL) for microarray analysis using 35,000-gene rat CodeLink oligonucleotide microarrays. Biotin-labeled cRNA was prepared by linear amplification of the poly(A)+ RNA population within the total RNA sample. Briefly, 100–500 pg of total RNA for each sample (quantified using Agilent Bioanalyzer) was amplified using the Arcturus RiboAmp HS kit (Molecular Devices). After a second round of cDNA synthesis and purification of double-stranded cDNA, *in vitro* transcription was performed using T7 RNA polymerase in the presence of biotinylated UTP. 10 µg of purified cRNA was fragmented to uniform size and applied to CodeLink Bioarrays (GE Healthcare) in hybridization buffer. Arrays were hybridized at 37°C for 20 h in a shaking incubator. Arrays were washed in 0.75× TNT at 46°C for 1 h and stained with Cy5-Streptavidin dye conjugate for 30 min. Dried arrays were scanned with a GenePix™ 4000B scanner.

Each biological replicate sample was hybridized to duplicate arrays. Thus, a total of 32 arrays representing 4 and 24 h time points after TBI were processed with CodeLink Expression Analysis software (GE Healthcare), and data were normalized, filtered, and queried for gene ontology category enrichment with GeneSpring software (Agilent Technologies). To compare individual expression values across arrays, raw intensity data from each probe (generated from CodeLink Expression 4.0 software) was normalized to the median intensity of the array. Only genes with normalized expression values greater than background intensity in at least one condition were used for further analysis. The starting gene list was further limited by only including genes with technical and sample replicate values having less than 2-fold variation. The resulting qualified gene list was used in individual treatment comparisons to determine genes that were differentially expressed across treatments.

### Data analysis

#### Neuronal Counting

The numbers of FJ-positive neurons were quantified for each of the four treatment groups and reported as mean +/− SEM and analyzed using analysis of variance (ANOVA) followed by the Bonferroni-Dunn test with α = 0.05. Statistical computations were carried out using PROC GLM in SAS®, Release9.1 [31].

#### Microarray Analysis

Gene expression levels in hippocampal neurons (considered both uninjured and surviving) of sham-injured rats were measured to establish baseline data for comparison of TBI-induced changes in known signaling pathways. All drug-induced gene expression changes in surviving hippocampal neurons were compared to mean gene expression levels in surviving neurons of TBI alone. Both TBI-induced changes >2-fold compared to the sham-injured baseline controls and the drug-treated samples were analyzed in GeneSpring for significant enrichment (hypergeometric p-values <0.05) of Gene Ontology categories as defined by the GO Consortium. All sham-injury and drug-induced gene expression changes >2-fold compared with TBI alone were uploaded into Ingenuity Pathway Analysis software (Ingenuity Systems, Redwood City, CA) and mapped to the functional networks available in the Ingenuity Pathway Knowledge Base. Canonical pathway analysis identified the pathways from the Ingenuity Pathways Analysis library of canonical pathways that were most significant to the data set. The significance of the association between the data set and the canonical pathway was measured (in IPA) in two ways: 1) A ratio of the number of molecules from the data set that map to the pathway divided by the total number of molecules that map to that pathway. 2) The right-tailed Fisher's exact test was used to calculate a *P*-value determining the probability that the association between the genes in the dataset and the canonical pathway is explained by chance alone. That is, over-represented functional or canonical pathways are processes which have more focus genes than expected by chance (right-tailed).

## Results

### Metyrapone and carbenoxolone reduce neurodegeneration in the rat hippocampus after TBI

**Figure 1:**
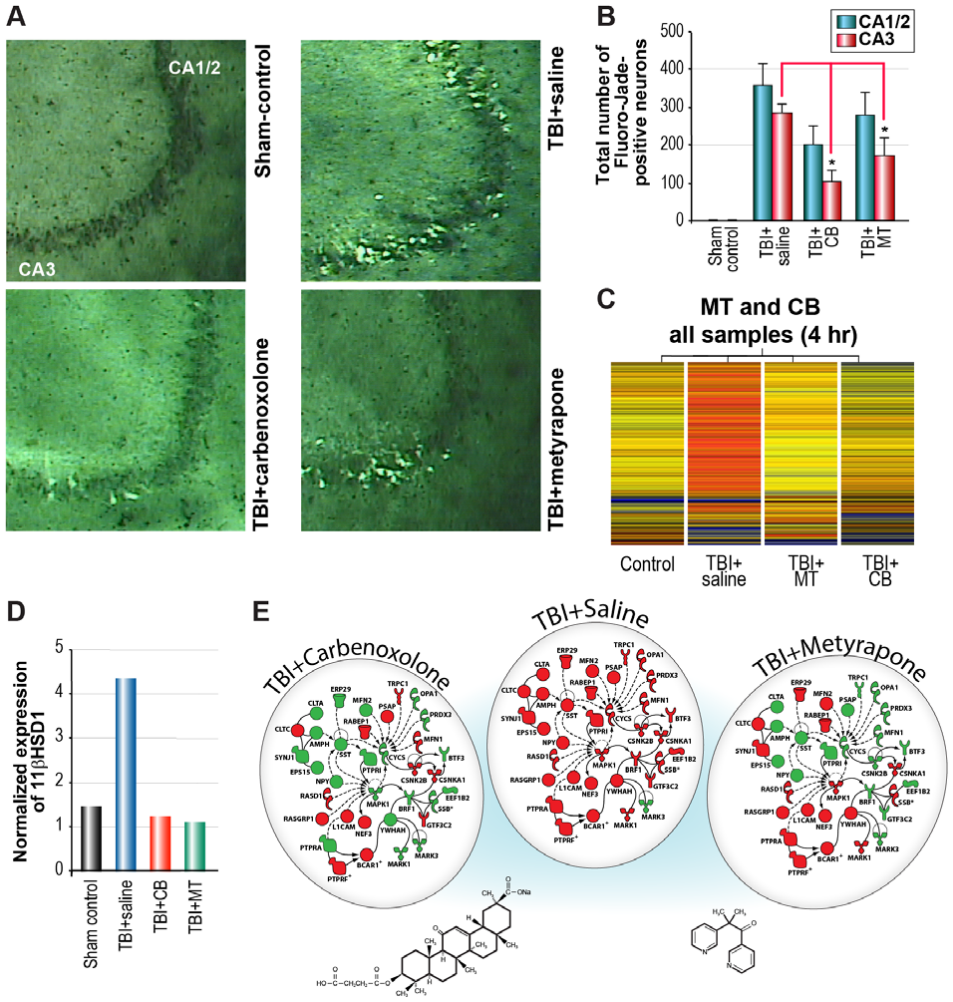
Neuroprotective effects of metyrapone (MT) or carbenoxolone (CB) treatment after traumatic brain injury (TBI). (**A,B**) Treatment of TBI rats with MT or CB significantly reduced numbers of degenerating, FJ-positive neurons in the CA3 subfield of the rat hippocampus. n = 6 per group, *p<0.05 (Bonferroni-Dunn test), CA (Cornu Ammonis). (**C**) Heat map of 870 genes >2-fold showing that both MT and CB treatment attenuated TBI-induced gene expression in hippocampal CA3 pyramidal neurons, in many cases returning gene expression levels to that of sham-operated control animals. (**D**) Both MT and CB prevented the TBI-induced increase in expression of 11βHSD1 (11β hydroxysteroid dehydrogenase type 1) in surviving, Fluoro-Jade-negative hippocampal neurons. (**E**) Although very different compounds, the transcriptional profiles induced by MT or CB are remarkably similar. Both drugs attenuate expression of several common genes in a custom pathway of TBI-induced genes. (See Fig. S15 for symbol key).

Rats were pretreated with MT or CB 30 min before TBI, and neurodegeneration in the hippocampus was evaluated 24 h post-injury using FJ staining [30], [32]. In the CA3 subfield of the injured hippocampus, both drug treatments significantly reduced the numbers of FJ-positive neurons (*P*<0.05 TBI vs. TBI+CB; *P*<0.05 TBI vs. TBI+MT). However, the differences did not reach statistical significance in the CA1/2 subfield of the hippocampus (Fig. 1A, B). These results demonstrate, for the first time, that both drugs reduce the number of degenerating neurons after fluid percussion TBI in a brain region critical for learning and memory.

### Similar effects of metyrapone and carbenoxolone on injury-induced gene expression

To address the hypothesis that MT and CB act via a common set of genomic targets, we used LCM to collect 600 FJ-negative (surviving) CA3 hippocampal neurons from rats from each of the four treatment groups (Sham, TBI, TBI+MT, TBI+CB). Total RNA was pooled for each treatment group and microarray analysis was performed using rat CodeLink BioArrays (GE Healthcare). At 4 h after TBI, a total of 870 genes were found to have >2-fold differential expression in either Sham, TBI+MT, or TBI+CB relative to TBI alone. The drug-treated samples had broadly similar effects in gene expression at 4 h. Both MT and CB attenuated injury-induced increases in gene expression at 4 h after TBI (Fig. 1C), and prevented the injury-induced increase in mRNA expression for 11βHSD1 (Fig. 1D). We found that the effects of both drugs at 24 h, with some exceptions, were more divergent, i.e., fewer common gene targets were affected by both drugs at this later time point, which prompted us to focus on common drug effects at 4 h.

Gene Ontology enrichment analysis revealed diverse biological processes and functions enriched with the 870 genes >2-fold at 4 h after TBI (Table S1). To gain further insight into the common neuroprotective mechanisms among these drugs, we decided to examine the effects of these drugs on known cell signaling pathways associated with cell survival or cell death. In these selected pathways, we performed *in silico* analysis of the functional roles of genes whose expression was altered in the same direction, downregulated or upregulated, by both drugs, and found that most of these commonly affected gene targets were associated with cell survival or neurodegenerative processes (References S1).

### Metyrapone and carbenoxolone treatments attenuate expression of genes in cell death pathways

We used Ingenuity Pathway Analysis (Ingenuity Systems, www.ingenuity.com) to focus on canonical cell signaling pathways affected by both drugs. The pathways we selected for further study were chosen primarily for their significance after IPA analysis, i.e., they represented the top pathways associated with the highest numbers of differentially expressed genes affected by both drugs. Representative examples of functional and canonical pathway analysis of differentially expressed genes in one comparison, TBI vs C, through IPA is shown in Fig. S1 and Tables S2, S3. This analysis was repeated for all comparisons. However, additional canonical pathways were also examined because of their biological relevance, as focusing on only statistically significant pathways may lead us to ignore pathways in which subtle, but biologically significant, changes are induced by both drugs. For instance, we examined the effects of both drugs on genes involved in CREB signaling, which is a key pathway associated with cell survival, but the fold changes in commonly affected genes were less than 2-fold, which may have eliminated this pathway from further analysis based on significance alone.

**Figure 2:**
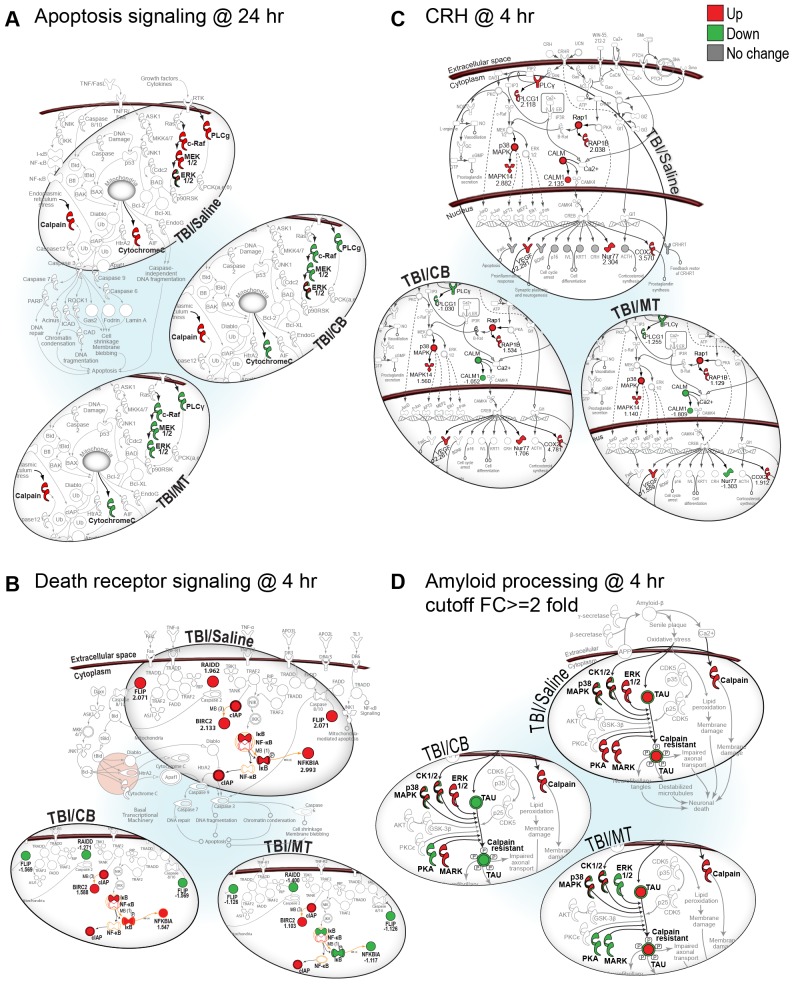
Common genes in cell death and stress response networks are affected by metyrapone or carbenoxolone. (**A**) Apoptosis signaling at 24 h post-TBI. (**B**) Death receptor signaling at 4 h post-TBI. (**C**) Corticotropin-releasing hormone (CRH) signaling at 4 h post-TBI. (**D**) Amyloid processing pathway at 4 h post-TBI. Gene expression in all groups for pathways A–C is shown up or down-regulated relative to sham control levels with no fold cut-offs. Metyrapone and carbenoxolone attenuate expression of both pro-death and pro-survival genes in these pathways such as FLIP, which is known to protect against ischemic cell death. (See Fig. S15 for symbol key).

We found a common subset of injury-induced genes that were up-regulated in the TBI group but down-regulated in both the TBI+MT and TBI+CB groups (custom pathway shown in Fig. 1E). In fact, both drugs attenuated expression of genes in canonical pathways associated with apoptosis and death receptor signaling (Fig. 2A,B. Note: All pathways are shown enlarged in Supporting information Figures S2, FS3, FS4, FS5, FS6, FS7, FS8, FS9, FS10, FS11, FS12, FS13, S14). In concordance with their effects on 11βHSD1, both MT and CB reduced expression of genes in the corticotrophin releasing hormone (CRH) pathway, which is associated with stress signaling (Fig. 2C). Examination of multiple canonical pathways showed that even when the specific gene targets of the two drugs were different, as in the amyloid processing pathway, the affected genes were members of the same canonical pathway (Fig. 2D).

### Metyrapone and carbenoxolone induce similar changes in cell survival pathways

**Figure 3:**
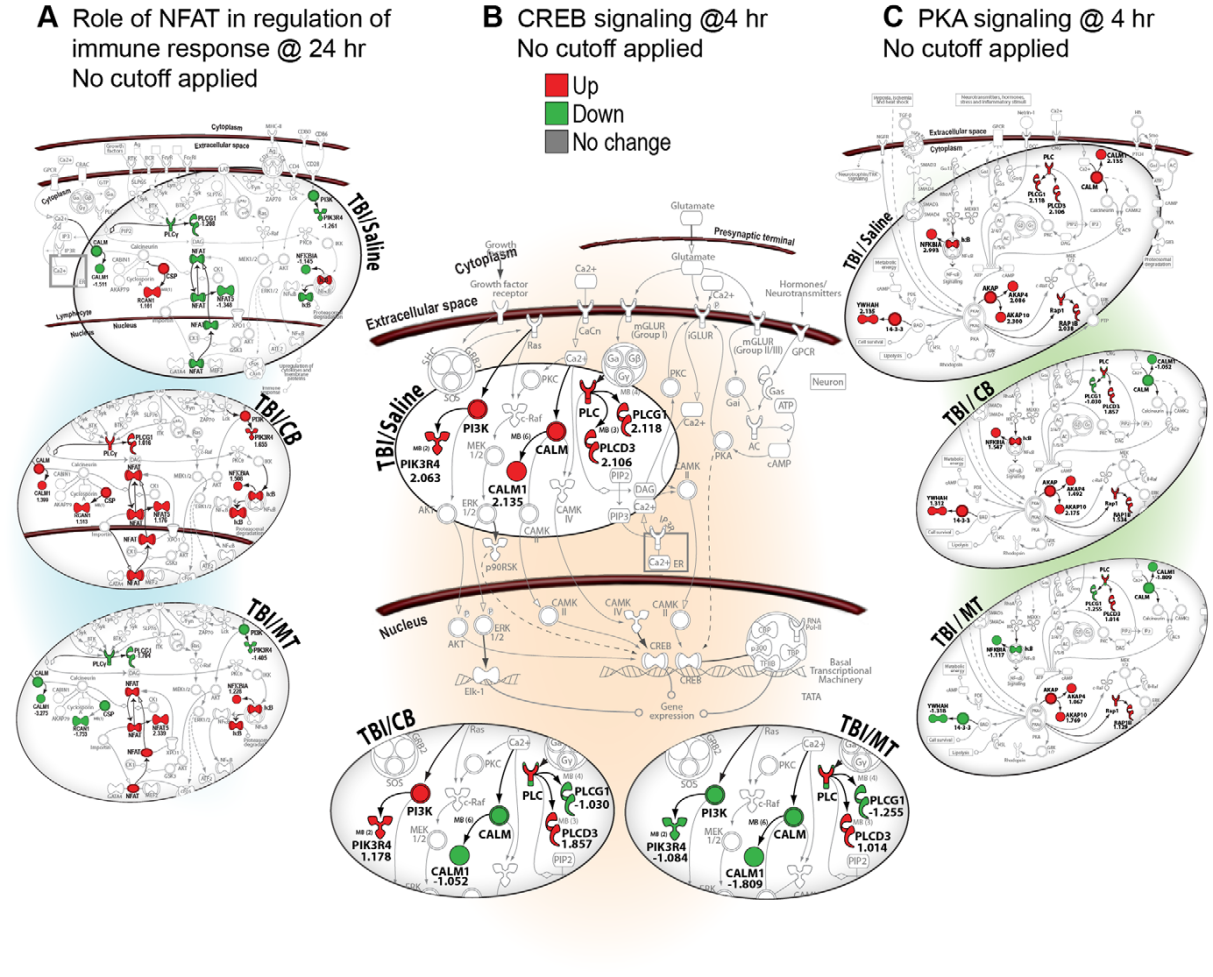
Metyrapone and carbenoxolone exert similar effects on several common gene targets in prosurvival pathways. (**A**) Nuclear factor of activated T cells (NFAT) signaling at 24 h post-TBI. (**B**) Cyclic AMP-response element binding (CREB) protein signaling at 4 h post-TBI. (**C**) Protein kinase A (PKA) signaling at 4 h post-TBI. These signaling pathways are associated with cell survival, synaptic plasticity, development and regeneration. (See Fig. S15 for symbol key).

**Figure 4:**
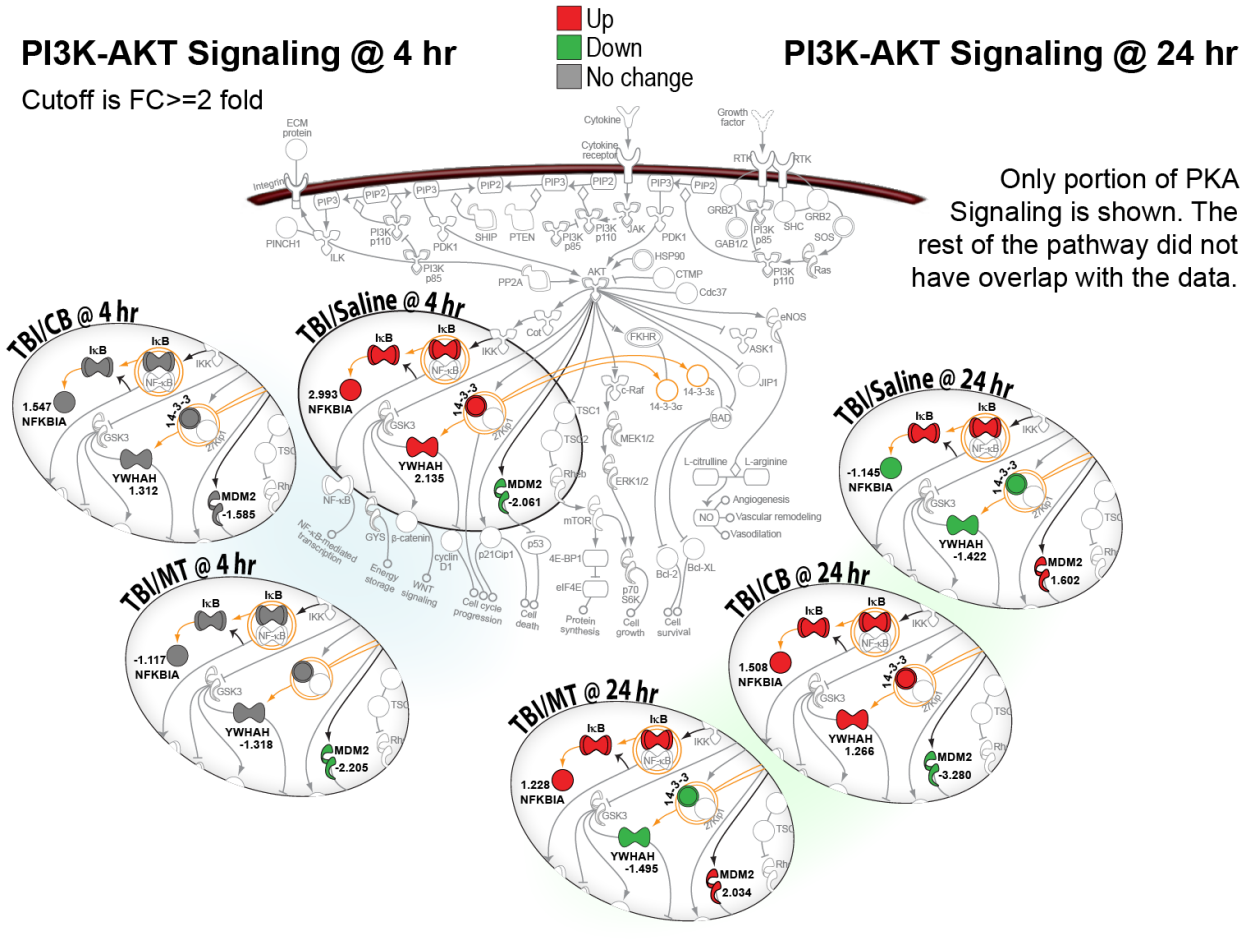
Metyrapone and carbenoxolone have similar effects on the pro-survival Phosphatidylinositol 3 kinase/AKT (PI3K/AKT) pathway. Gene expression is shown at 4 h (2 fold cut-off) and 24 h post-TBI (no cut-off). A key cell survival associated gene, NFκB1A, is upregulated by both drugs at 24 h compared with TBI alone. (See Fig. S15 for symbol key).

**Figure 5:**
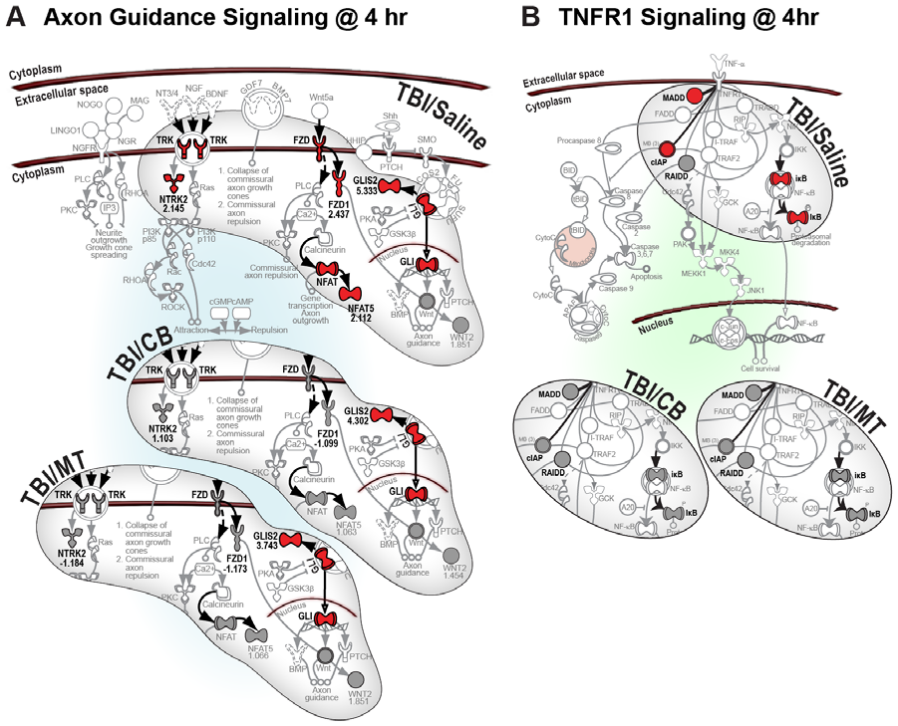
Metyrapone and carbenoxolone attenuate gene expression in both pro-survival and pro-death pathways. (**A**) Axon guidance signaling pathway is associated with regeneration and growth. (**B**) TNFR1 signaling pathway is associated with inflammation and cell death. (See Fig. S15 for symbol key).

We found that both MT and CB increased expression of genes in the NFAT signaling pathway after TBI (Fig. 3A). Moreover, we also found that they decreased expression of common genes in the CREB and PKA pathways (Figure 3B, C). Interestingly, the NFAT, CREB and PKA signaling pathways are all associated with cell survival and regeneration [33], [34], [35]. We also observed that temporal changes in gene transcription were similarly affected by the two drugs in the PI3K/AKT cell survival pathway; increased expression of survival-associated genes was noticeably absent after treatment with either drug 4 h post-injury. However, 24 h post-TBI, when some of the injury-induced genes in this survival pathway were downregulated in untreated TBI rats, drug treatment increased expression of several genes previously downregulated at 4 h (Fig. 4), suggesting a delayed induction of survival signaling. Most prominently, we repeatedly observed that drug treatments attenuated or normalized injury-induced gene expression regardless of whether the cell signaling pathways were associated with cell proliferation, growth and survival (e.g., axon guidance) or inflammation and cell death (e.g., TNFR1), (Fig. 5A, B).

### Metyrapone and carbenoxolone attenuate expression of genes associated with generation of reactive oxygen species (ROS)

**Figure 6:**
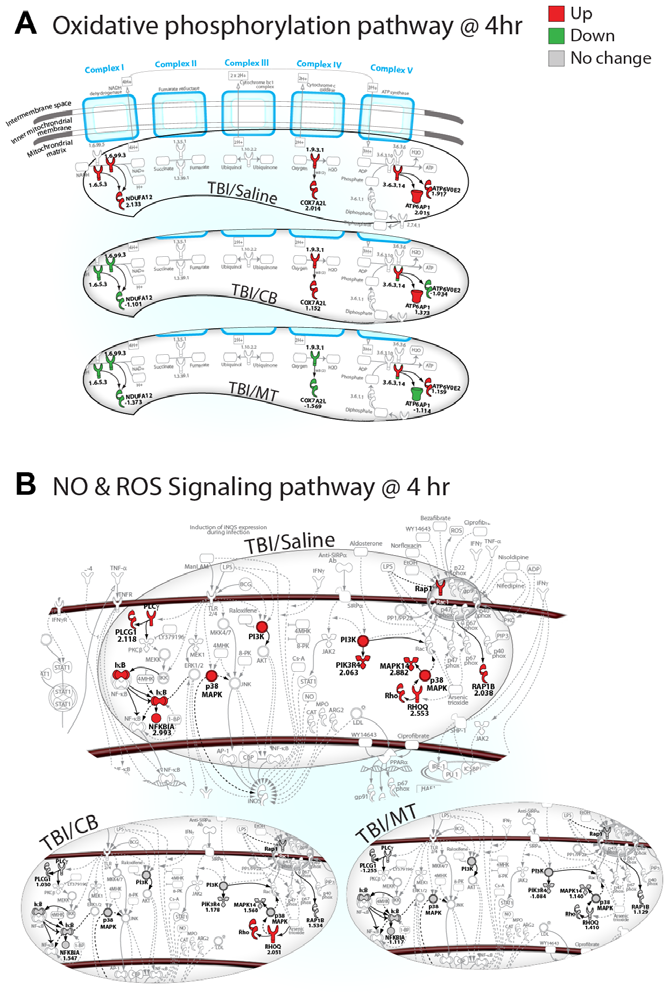
Metyrapone and carbenoxolone attenuate gene expression in pathways associated with production of reactive oxygen species (ROS). (**A**) Both drugs downregulate expression of subunits of NADH dehydrogenase in Complex 1 (primary source of reactive oxygen species produced in mitochondria), cytochrome c oxidase (Complex IV) and ATP synthase (Complex V) in the mitochondrial oxidative phosphorylation pathway at 4 h post-TBI. (**B**) Expression of multiple genes in the canonical Nitric oxide and Reactive Oxygen Species (NO & ROS) in macrophages pathway at 4 h post-TBI (2 fold cut-off) are also downregulated after metyrapone or carbenoxolone treatment suggesting a drug-induced reduction in oxidative stress. (See Fig. S15 for symbol key).

Both MT and CB altered metabolic pathways linked to oxidative stress. Although neurodegenerative diseases are associated with mitochondrial dysfunction, drugs that induce a mild uncoupling of oxidative phosphorylation (OxPhos), i.e., decrease the proton gradient and mitochondrial potential (ΔΨ) [36], have neuroprotective properties [37], [38]. MT and CB attenuated expression of genes in the OxPhos (Fig. 6A) and nitric oxide/reactive oxygen species (NO/ROS) pathways (Fig. 6B), particularly genes coding for subunits of NADH dehydrogenase (Complex 1), which produces most of the ROS in mitochondria [39]. This suggests that their protective effects are linked, in part, to reduction of ROS-induced cell death. Indeed, the overall effect of both drugs was to prevent the injury-induced upregulation of multiple genes in the OxPhos pathway; both drugs also attenuated expression of separate genes associated with subunits of cytochrome c oxidase (Complex IV) and ATP synthase (Complex V), suggesting an overall inhibition of mitochondrial function.

## Discussion

This is the first molecular comparison of the potential mechanisms underlying the neuroprotective effects of MT and CB, and of the molecular targets and associated cell signaling pathways that mediate their protective effects on hippocampal neurons after TBI. Previous reports have shown that, in addition to their nootropic effects and common gene targets, both MT and CB reduce neuronal injury both *in vitro* and *in vivo* [10], [11], [40]. These studies served as the primary rationale for the choice of these two structurally different compounds for our comparative pathway analysis. However, neither drug has previously been evaluated in a fluid-percussion TBI model [41].

The complex pathogenesis of TBI is associated with injury-induced changes in multiple molecular pathways [42], [43], making it difficult to evaluate novel therapeutic agents with multiple postulated mechanisms of action in pre-clinical animal models. Recent studies have shown that distinct molecular networks that underlie core biological processes can be used as biosensors or biomarkers for human diseases [44], and distinct genetic programs (coordinated activation and/or repression of genes in known cell signaling pathways) are associated with neurodegenerative diseases [45]. Considering these facts, we hypothesized that distinct alterations in a specific set of biological pathways associated with cell survival or cell death could serve as biomarkers of TBI pathology. Therefore, we reasoned that effective therapeutic drugs would alter expression of common genes in these specific injury-induced cell survival and/or cell death pathways. Moreover, in order to effectively alter physiological responses that regulate neuronal fate after TBI, effective neuroprotective agents will likely influence multiple components of a coordinated response. In this study, we used a systems biology approach to investigate drug-induced changes in a broad spectrum of biological pathways affected by TBI, albeit with a special focus on pathways associated with cell survival or cell death since our objective was to gain insight into the neuroprotective mechanisms of these drugs [4]. Recent reports using this approach have demonstrated that neurodegenerative diseases influence broad neuronal networks [46], and that distinct disease-modifying pathways can be associated with neurodegeneration [45]. This has led to insightful discoveries such as cessation of organ growth in adult rats being mediated by a defined genetic program that coordinates downregulation of multiple growth-promoting genes [47].

The main finding of our study is that a distinctive common molecular signature characterizes the effects of MT and CB, thus suggesting an avenue for investigation of the pathophysiology of TBI, as well as evaluation of potential treatment strategies for TBI. Our data show that the cumulative genomic response to these drugs appears to be a homeostatic improvement or normalization in injury-induced signaling. Specifically, MT and CB attenuate injury-induced gene expression associated with neurodegeneration and cell death, and, contrary to our expectation that these drugs would increase mobilization of protective genes, both drugs attenuate injury-induced gene expression associated with cell survival.

Another important outcome of our study is that we show, for the first time, that MT and CB reduce the number of degenerating neurons after TBI in the hippocampus, a brain region critical for learning and memory. These data are concordant with previous studies showing that these drugs prevented ischemia-induced synaptic dysfunction in the hippocampus [40], [48].

Because the goal of this study was to understand drug-induced neuroprotection, it was essential to examine gene expression in only neurons that survived TBI, without the contaminating data deriving from dying neurons. In an earlier study, LCM made it possible for us to selectively capture only surviving, FJ-negative pyramidal neurons from the CA3 subfield of the rat hippocampus at 24 h post-TBI [27]. In this previous study, by comparing changes in gene expression in surviving cells (FJ-negative) to those in non-surviving cells (FJ-positive) after TBI, we identified specific cell signaling networks known to play an essential role in injury response and survival. These data, followed by examination of the combined expression profiles of the pathways in which many (6 or more) co-expressed or functionally linked genes were altered by drug treatment, provided the most useful insight into the common mechanisms of these neuroprotective drugs. Our observation that both drugs similarly affected a distinct set of common target genes that were induced after TBI in these canonical pathways, suggests that altered expression of these specific genes was associated with their protective effects.

While it is impossible to predict whether the surviving neurons in untreated TBI rats would have died or survived at a later time point, we know that, at 24 h post TBI, the neurons analyzed in our study were not undergoing neurodegeneration as determined by FJ staining. However, even surviving neurons are expected to show a transcriptional profile reflecting their exposure to TBI in the absence of drug treatment, and surviving neurons from treated animals would show gene expression profiles influenced by drug treatment.

Whether or not the final outcome is death or survival, hippocampal neurons mount a protective molecular response after TBI. In the case of the dying neurons, the response is inadequate. This suggests that neuronal survival or death is regulated by a cell survival rheostat that is determined based on a ratio of cell survival to cell death genes that, in turn, depends on the stochastic expression of these genes in hippocampal neurons before TBI [27]. This stochastic component might mask small, not necessarily statistically significant albeit biologically significant, changes in expression induced by drug treatment. In many of these cell survival or cell death associated pathways, the common gene targets of both drugs only became evident when the 2-fold change cut-off was not applied and used to screen out certain pathways for further study. Indeed, by first selecting pathways based on biological relevance and then examining the alterations in gene components, we gained an insight into the neuroprotective mechanisms of MT and CB that we would have missed totally by only studying statistically significant pathways. Interestingly, the rheostat-like effects of small regulatory non-coding microRNAs on target mRNAs are often less than 2-fold, within the boundaries of random variations, but still biologically significant [49]. Our data support the concept that a cell survival rheostat could be influenced by drugs that induce subtle changes in individual gene expression, within co-expression networks, resulting in coordinated and biologically significant changes in cell signaling pathways. In many cases, the fold changes in gene expression induced by drug treatment were less than two-fold. However, the key observation we made after IPA analysis is that both CB and MT produced consistent and similar changes in co-expressed genes in dozens of canonical pathways associated with cell survival/plasticity/regeneration or cell death/stress/degeneration. Consequently, a coordinated pattern of significant gene expression changes only became apparent when the effect of these drugs was evaluated on entire cell signaling pathways rather than on individual genes with significant p-values.

Pathway analysis revealed several biologically relevant events. Specifically, acute and long-term neurodegeneration is the result of TBI-induced dysregulation of genes in multiple cell signaling networks, and neurons regulate synaptic plasticity by regulating the expression of activity-induced gene expression [50].Thus, our data suggest that CB and MT exert their neuroprotective effects via homeostatic normalization of injury-induced gene expression to pre-injury levels. Moreover, the similar transcriptional profiles induced by CB and MT support our hypothesis that neuroprotective drugs work through a common set of molecular targets associated with cell survival or cell death.

The attenuation or normalization of injury-induced gene expression is one of the defining molecular signatures in our study, suggesting that, paradoxically, neuroprotection is associated with an overall lack of mobilization of protective genes. Although we did observe upregulation of some survival-associated genes in the NFAT and PI3K pathways, the attenuating effects of both drugs on several pathways associated with cell death and stress signaling are also consistent with previous studies showing that improved levels of neuroprotection were obtained with therapeutic agents that targeted multiple pathways involved in cell death [51].

Both CB and MT display inhibitory effects on 11βHSD1, and, hence, glucocorticoid production, resulting in inhibition of glucocorticoid-regulated targets such as neuropeptide Y and tyrosine hydroxylase [52], [53]. However, pathway analysis confirmed our original hypothesis that the neuroprotective effects of MT and CB are mediated, in part, by common molecular targets other than 11βHSD1. In fact, many of the TBI-induced gene expression changes observed in our study have not been previously associated with glucocorticoid treatment.

Extensive *in silico* validation of genes affected by both compounds (GEO accession GSE31357, References S1) showed that many of the genes present in pathways affected by MT and CB treatment are associated with regenerative and/or pro-survival phenotypes, while others are associated with cell death signaling. A key feature of recovery after injury is the reactivation of cellular programs characteristic of embryological development [54], [55]. The CREB, NFAT, PKA and PI3K/AKT signaling pathways are associated with cell survival, but are also well known to be essential in development and regeneration. Expression of genes associated with tumorigenesis was down-regulated or normalized by both drugs (see References S1). Tumor cells often have the ability to invade, resist cell death-inducing signals, and metastasize to other regions of the body by activating developmental regulatory programs [56]. Therefore, a limited repertoire of cell survival signals appears to be common to both tumorigenesis and neuronal survival after TBI. Several genes in the amyloid processing pathway were also affected by MT and CB. This is particularly relevant because much evidence links early onset of Alzheimer's disease (AD) in TBI survivors to accumulation of amyloid-β (Aβ), due to a TBI-induced imbalance in Aβ genesis and catabolism [57].

Because TBI produces significant oxidative stress in the rat hippocampus [58], we also examined the effects of MT and CB on canonical pathways associated with generation of reactive oxygen species. ROS are byproducts of oxidative phosphorylation [39] and nitric oxide signaling [59], primarily in the mitochondria. The effects of both drugs on mitochondrial gene expression provided provocative insights, as previous studies have shown that the effects of glucocorticoids on mitochondrial function follow a U shaped curve (low doses are protective while higher doses are associated with suppression of Bcl-2 levels and decreases in neuronal survival) [60]. CB, but not MT, has been shown to uncouple mitochondrial OxPhos [61]. Here we showed, for the first time, that the overall effect of both drugs on genes involved in OxPhos would predict a reduction of ROS consistent with a mild uncoupling of OxPhos. Previous studies have shown that increased expression of uncoupling proteins decreases production of mitochondrial ROS [62]. Moreover, deletion of uncoupling protein 2 (UCP-2) in mice exacerbated ischemic brain injury, and was associated with suppression of cell repair genes and antioxidants [63].

Interestingly, compounds that partially inhibit OxPhos can increase tolerance to subsequent hypoxia [64]. Because compounds that reduce mitochondrial oxidant stress, such as the L-type calcium channel blockers, nicardipine and nimopidine, also have been shown to have neuroprotective effects, they have been proposed as treatments for neurodegenerative disorders [65]. Nonsteroidal, anti-inflammatory drugs also uncouple OxPhos [66], which suggests an additional mechanism for their neuroprotective effects in neurodegenerative disorders such as AD [67], [68].

Finally, one proposed neuroprotective strategy involves reduction of oxidative stress using therapeutic agents such as resveratrol [69] or interventions such as caloric restriction [70]. Resveratrol has been shown to exert its neuroprotective effects by a cell signaling pathway involving UCP2 [71], suggesting that, in general, other uncouplers of OxPhos have therapeutic potential for TBI. In line with this, a recent study suggested that novel protective drugs could be discovered by their ability to shift energy metabolism from mitochondrial respiration to glycolysis, which is known to suppress oxidative stress and cell death after ischemia [72].

Although the undesirable side-effect profiles of these drugs preclude long-term use in TBI patients, the demonstration that, following a single treatment, MT or CB reduces neurodegeneration, indicates that acute, short-term treatment after TBI might safely mitigate some of the deleterious effects of the initial injury. These two drugs associated with neuroprotection and cognitive enhancement induce a distinctive molecular profile associated with a reduction in oxidative stress and cell death. Additionally, many drugs in clinical use for other purposes may exhibit similar effects. Thus, the screening of such drugs for efficacy in TBI patients is warranted. Drugs identified using these criteria may also be beneficial for other neurodegenerative disorders in which similar cell survival pathways are affected.

### Accession codes

Microarray data is MIAMI compliant and has been deposited in the Gene Expression Omnibus under the accession number GSE31357.

## Supporting Information

Figure S1 **Representative example of functional and canonical pathway analysis in IPA.** Top functions, processes and pathways with the greatest numbers of differentially expressed genes across experimental treatment groups were identified for further analysis. Bar graph of top pathways in the TBI vs Control comparison at 4 h are shown. Higher bars denote greater significance and the numbers of genes above the yellow line, which signifies a p value of 0.05, represent those that are significantly differentially expressed than by chance alone.
(PDF)

Figure S2 **Ingenuity pathway analysis showing the effects of carbenoxolone or metyrapone treatment on a custom pathway of injury-induced genes.** The drug-induced profiles are remarkably concordant. i.e., the drugs attenuated expression of multiple, common genes that were upregulated after TBI. (See Fig. S15 for symbol key).
(PDF)

Figure S3 **Ingenuity pathway analysis showing the effects of TBI and drug treatment on the canonical apoptosis signaling pathway at 24 h post-TBI.** Key cell signaling genes associated with apoptosis are commonly downregulated by carbenoxolone or metyrapone. (See Fig. S15 for symbol key).
(PDF)

Figure S4 **Ingenuity pathway analysis of canonical death receptor signaling pathway at 4 h post-TBI.** Both metyrapone and carbenoxolone attenuate expression of FLIP, which is known to protect against ischemic cell death. This suggests that drug treatment obviated the need for a protective response. (See Fig. S15 for symbol key).
(PDF)

Figure S5 **Ingenuity pathway analysis of canonical corticotrophin-releasing hormone (CRH) signaling pathway at 4 h post-TBI.** Both metyrapone and carbenoxolone attenuate expression of key cell signaling intermediates. (See Fig. S15 for symbol key).
(PDF)

Figure S6 **Ingenuity pathway analysis of canonical amyloid processing pathway at 4 h post-TBI.** Although different genes are affected by metyrapone or carbenoxolone, it is noteworthy that these drugs affect a signaling pathway linked to Alzheimer's disease for which TBI is a risk factor. Gene expression data are shown with a 2-fold cut off. (See Fig. S15 for symbol key).
(PDF)

Figure S7 **Ingenuity pathway analysis of canonical cyclic AMP response element binding (CREB) protein signaling pathway at 4 h post-TBI.** Both metyrapone and carbenoxolone attenuate common genes in this cell survival pathway—a theme that is repeatedly observed in many other injury-induced cell signaling pathways. (See Fig. S15 for symbol key).
(PDF)

Figure S8 **Ingenuity pathway analysis of NFAT in regulation of immune response pathway at 24 h post-TBI.** Similar to the PI3K/AKT pathway (also see Figure S10b), cell survival-associated genes downregulated after TBI alone are upregulated by metyrapone or carbenoxolone treatment 24 h after injury. (See Fig. S15 for symbol key).
(PDF)

Figure S9 **Ingenuity pathway analysis of canonical protein kinase A (PKA) signaling pathay at 4 h post-TBI.** Again, both metyrapone and carbenoxolone attenuate expression of key cell signaling intermediates associated with cell survival. (See Fig. S15 for symbol key).
(PDF)

Figure S10 **Ingenuity pathway analysis of canonical phosphotidylinositide 3 kinase/AKT (PI3K/AKT) signaling pathway with 2-fold cut off** (**A**) This illustrates that both metyrapone and carbenoxolone attenuate expression of injury-induced genes in cell survival pathways at 4 h post-TBI. (**B**) A key cell survival associated gene, NFκB1A is upregulated by both metyrapone and carbenoxolone at 24 h post-TBI compared with TBI alone. (See Fig. S15 for symbol key).
(PDF)

Figure S11 **Ingenuity pathway analysis of axon guidance pathway at 4 h post-TBI.** Metyrapone and carbenoxolone attenuate expression of common genes associated with cell survival and regeneration in this pathway. (See Fig. S15 for symbol key).
(PDF)

Figure S12 **Ingenuity pathway analysis of TNFR1 signaling pathway at 4 h post-TBI with 2-fold cut off.** Metyrapone and carbenoxolone attenuate expression of common genes associated with cell death and inflammation. (See Fig. S15 for symbol key).
(PDF)

Figure S13 **Ingenuity pathway analysis of canonical oxidative phosphorylation pathway at 4 h post-TBI.** Genes coding for NADH dehydrogenase, the first enzyme in Complex 1 of the mitochondrial electron transport chain are downregulated by both metyrapone and carbenoxolone. This is the site of production of most of the reactive oxygen species in the inner mitochondrial membrane. (See Fig. S15 for symbol key).
(PDF)

Figure S14 **Ingenuity Pathway Analysis of nitric oxide and reactive oxygen species (NO&ROS) signaling pathway in macrophages at 4 h post-TBI with 2-fold cut off.** Multiple injury-induced genes in this pathway are downregulated by metyrapone and carbenoxolone treatment compared to TBI alone. (See Fig. S15 for symbol key).
(PDF)

Figure S15 **Symbol key for pathways.**
(PDF)

Table S1 **Gene Ontology.** Genes affected by metyrapone and carbenoxolone treatment participate in diverse cell signaling processes.
(PDF)

Table S2 **Pathways with Highest # Gene Overlap (partial list).**
(PDF)

Table S3 **Top significant pathways of interest with biological relevance.**
(PDF)

References S1 Supplementary References
(PDF)
