# Supplementary figures and images for: Pathway Analysis Reveals Common Pro-Survival Mechanisms of Metyrapone and Carbenoxolone after Traumatic Brain Injury
Source: PLoS One. 2013 Jan 9;8(1):e53230. doi: 10.1371/journal.pone.0053230 (PMC3541279; doi:10.1371/journal.pone.0053230)

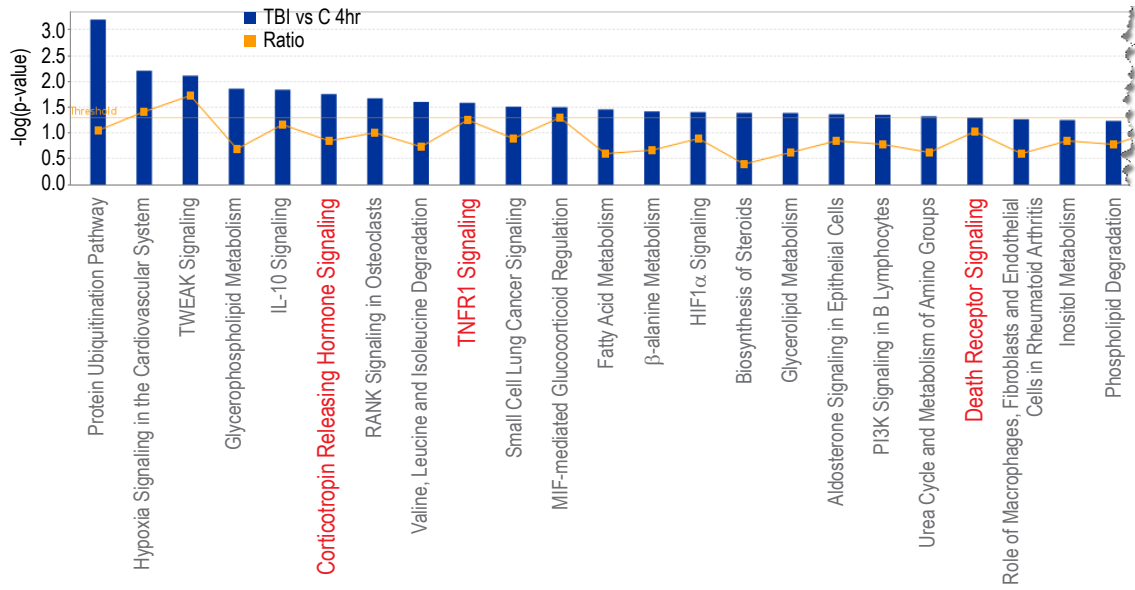

Genes: FC > 2-fold

Pathways: Threshold = pvalue 0.05

Supplement: Figure S1 — Representative example of functional and canonical pathway analysis in IPA. Top functions, processes and pathways with the greatest numbers of differentially expressed genes across experimental treatment groups were identified for further analysis. Bar graph of top pathways in the TBI vs Control comparison at 4 h are shown. Higher bars denote greater significance and the numbers of genes above the yellow line, which signifies a p value of 0.05, represent those that are significantly differentially expressed than by chance alone. (PDF) [file pone.0053230.s001.pdf]

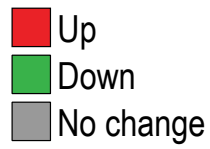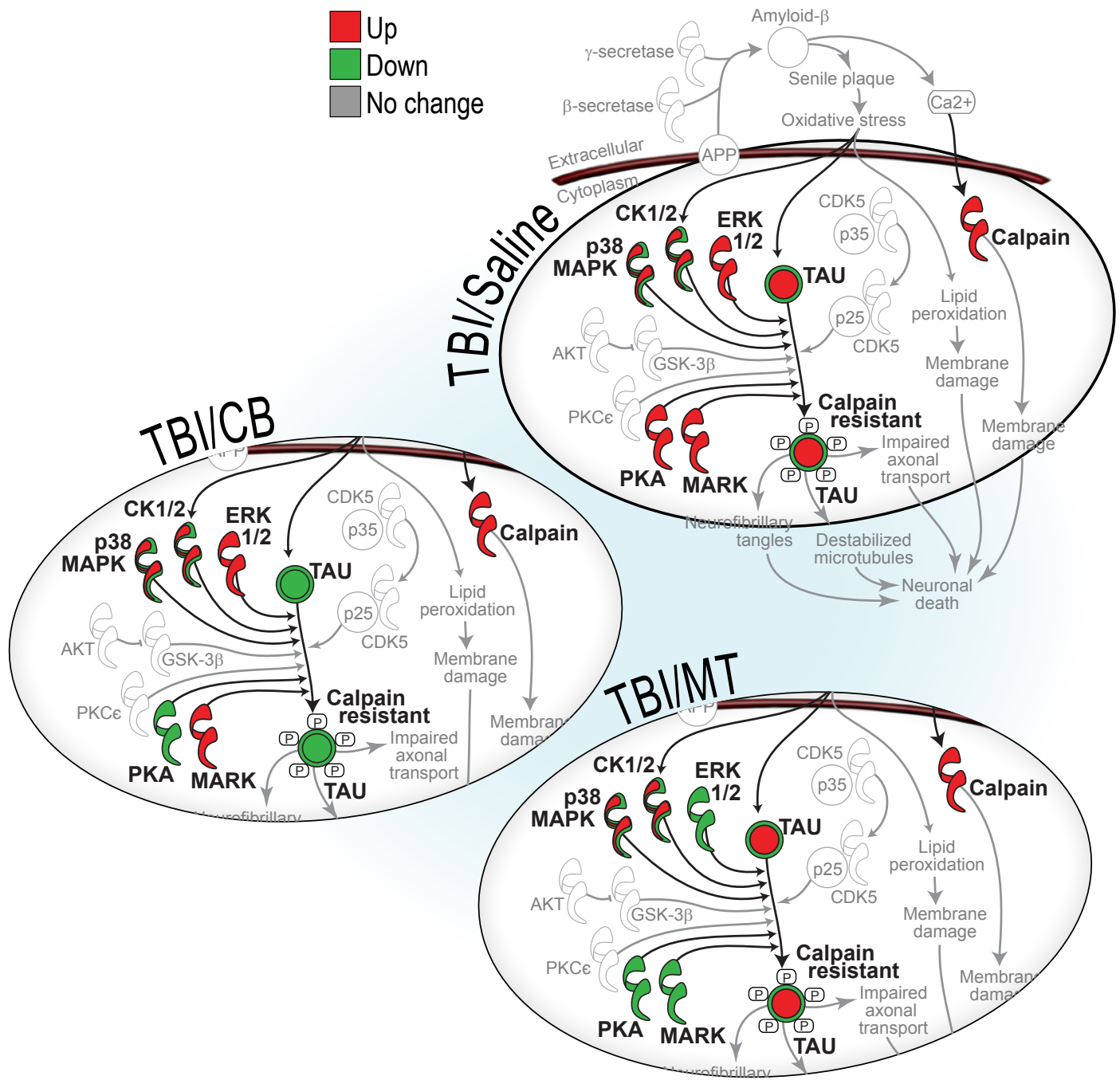

Supplement: Figure S6 — Ingenuity pathway analysis of canonical amyloid processing pathway at 4 h post-TBI. Although different genes are affected by metyrapone or carbenoxolone, it is noteworthy that these drugs affect a signaling pathway linked to Alzheimer's disease for which TBI is a risk factor. Gene expression data are shown with a 2-fold cut off. (See Fig. S15 for symbol key). (PDF) [file pone.0053230.s006.pdf]

■ Up  
■ Down  
■ No change

Extracellular space  
Cytoplasm

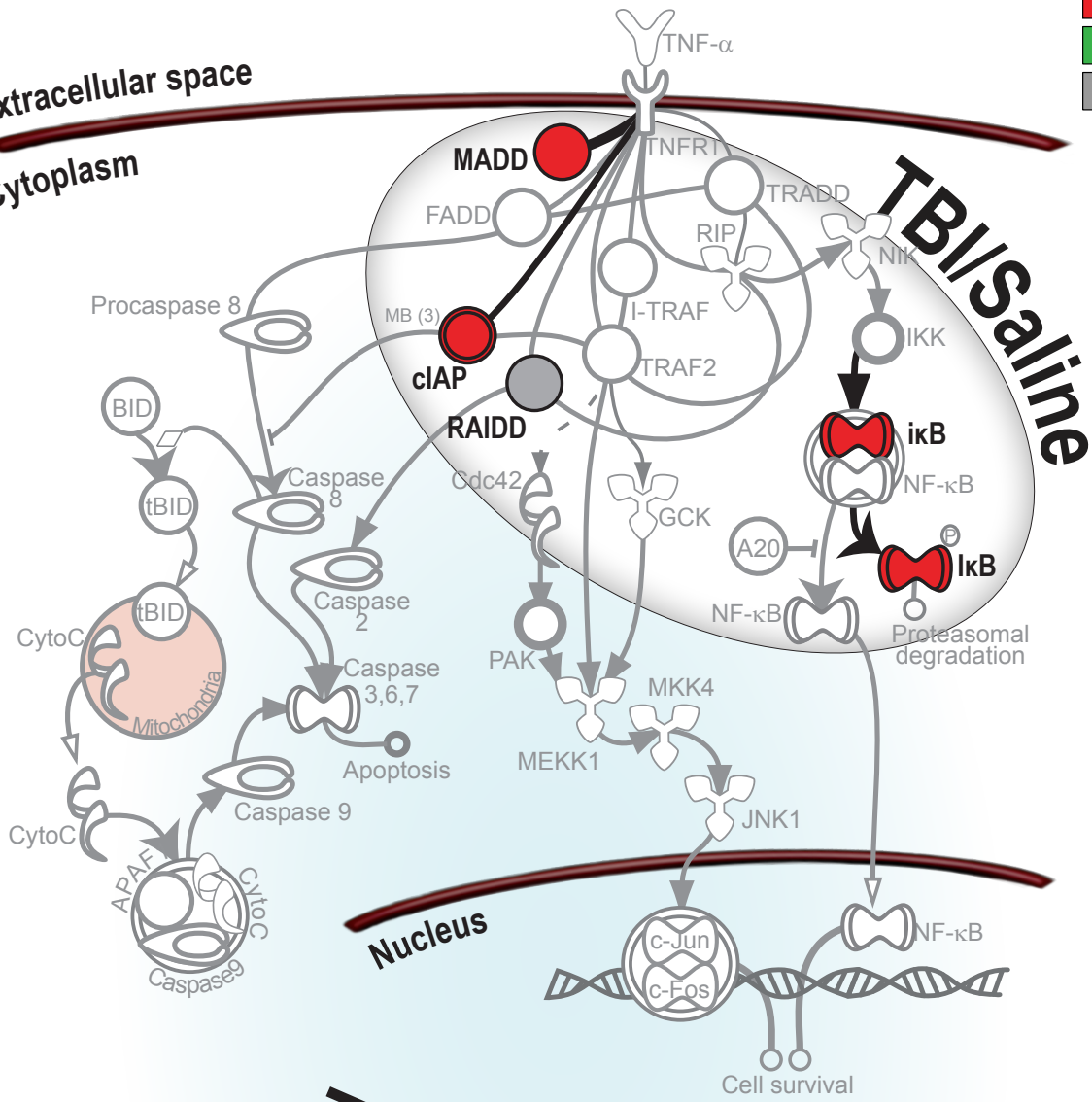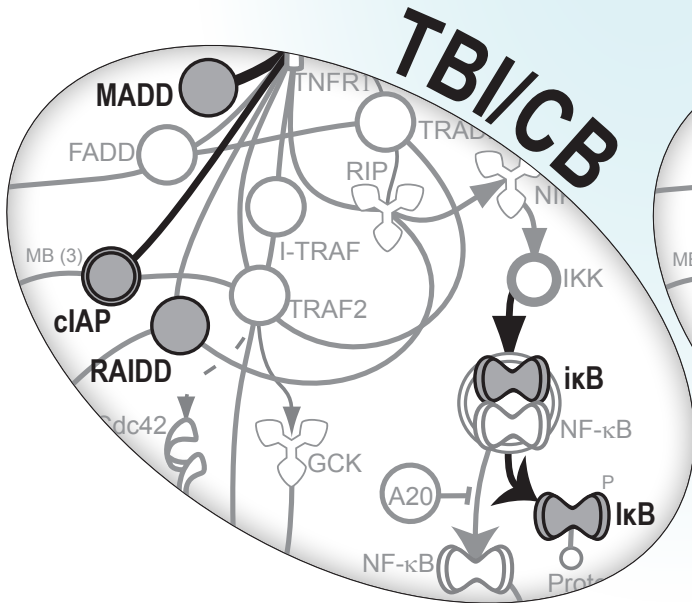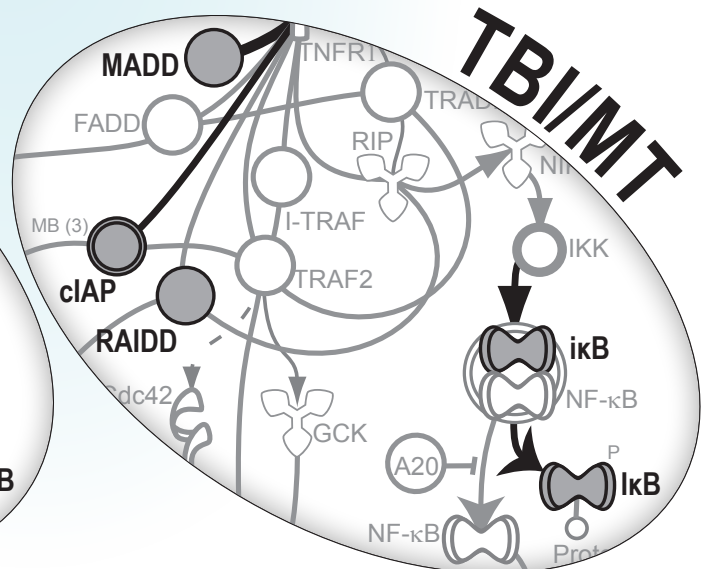

Supplement: Figure S12 — Ingenuity pathway analysis of TNFR1 signaling pathway at 4 h post-TBI with 2-fold cut off. Metyrapone and carbenoxolone attenuate expression of common genes associated with cell death and inflammation. (See Fig. S15 for symbol key). (PDF) [file pone.0053230.s012.pdf]
