# Supplementary material for: Pathway Analysis Reveals Common Pro-Survival Mechanisms of Metyrapone and Carbenoxolone after Traumatic Brain Injury
Source: PLoS One. 2013 Jan 9;8(1):e53230. doi: 10.1371/journal.pone.0053230 (PMC3541279; doi:10.1371/journal.pone.0053230)
Supplement: Figure S2 — Ingenuity pathway analysis showing the effects of carbenoxolone or metyrapone treatment on a custom pathway of injury-induced genes. The drug-induced profiles are remarkably concordant. i.e., the drugs attenuated expression of multiple, common genes that were upregulated after TBI. (See Fig. S15 for symbol key). (PDF) [file pone.0053230.s002.pdf]

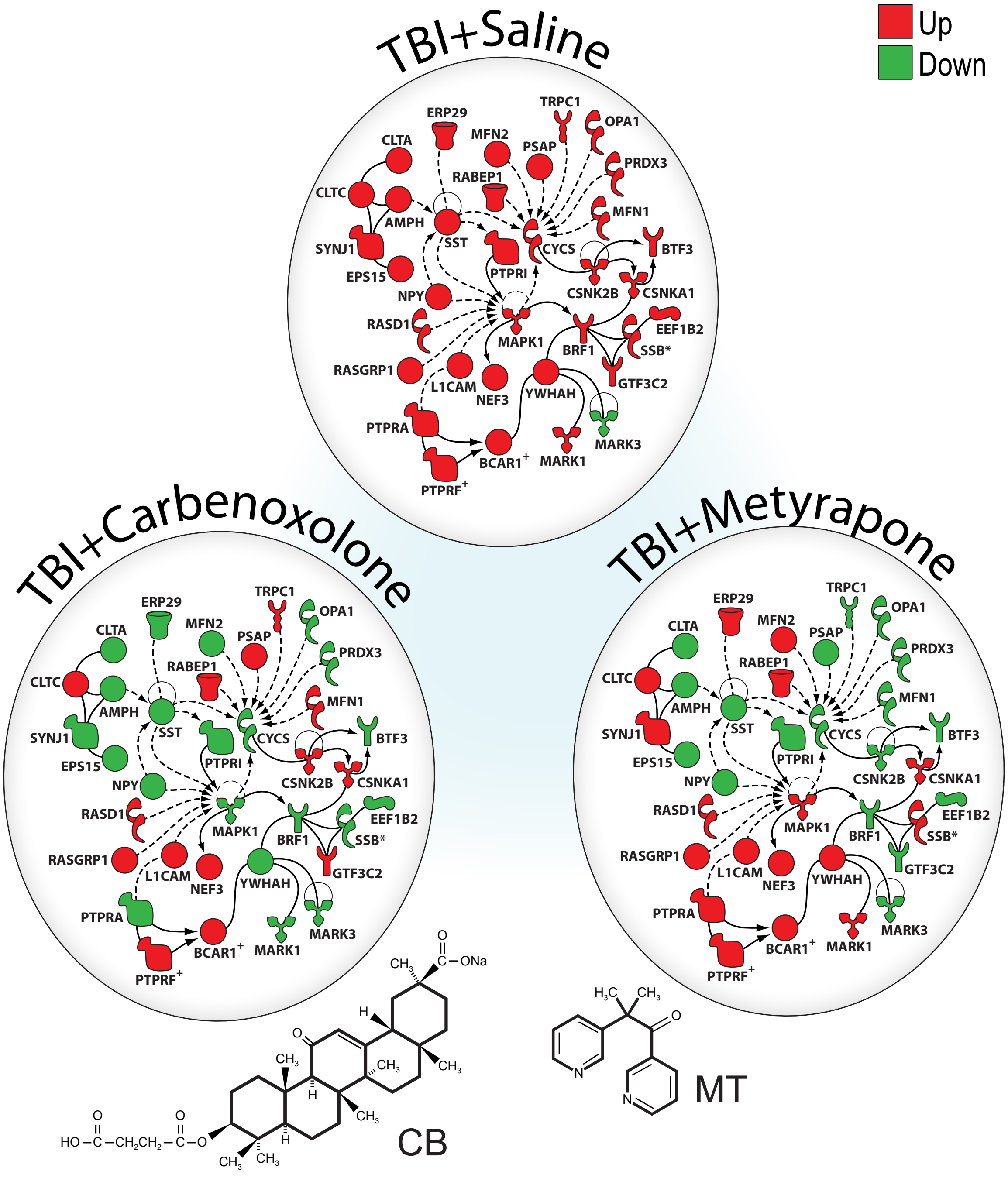

CLTA [S1,S2]	Clathrin, light chain A
EPS15 [S3,S4]	Epidermal growth factor receptor substrate 15
AMPH [S5,S6]	Amphiphysin
SST [S7,S8]	Somatostatin
NPY [S9,S10]	Neuropeptide Y
BRF1 [S11]	BRF1 homolog, subunit of RNA polymerase III transcription initiation factor IIIB (S. cerevisiae)
EEF1B2 [S12]	Eukaryotic translation elongation factor 1 beta 2
BTF3 [S13,S14]	Basic transcription factor 3
PRDX3 [S15,S16]	Peroxiredoxin 3
OPA1 [S17]	Optic atrophy 1
PTPR1 [S18]	protein tyrosine phosphatase receptor 1
