# Supplementary material for: Pathway Analysis Reveals Common Pro-Survival Mechanisms of Metyrapone and Carbenoxolone after Traumatic Brain Injury
Source: PLoS One. 2013 Jan 9;8(1):e53230. doi: 10.1371/journal.pone.0053230 (PMC3541279; doi:10.1371/journal.pone.0053230)
Supplement: Figure S3 — Ingenuity pathway analysis showing the effects of TBI and drug treatment on the canonical apoptosis signaling pathway at 24 h post-TBI. Key cell signaling genes associated with apoptosis are commonly downregulated by carbenoxolone or metyrapone. (See Fig. S15 for symbol key). (PDF) [file pone.0053230.s003.pdf]

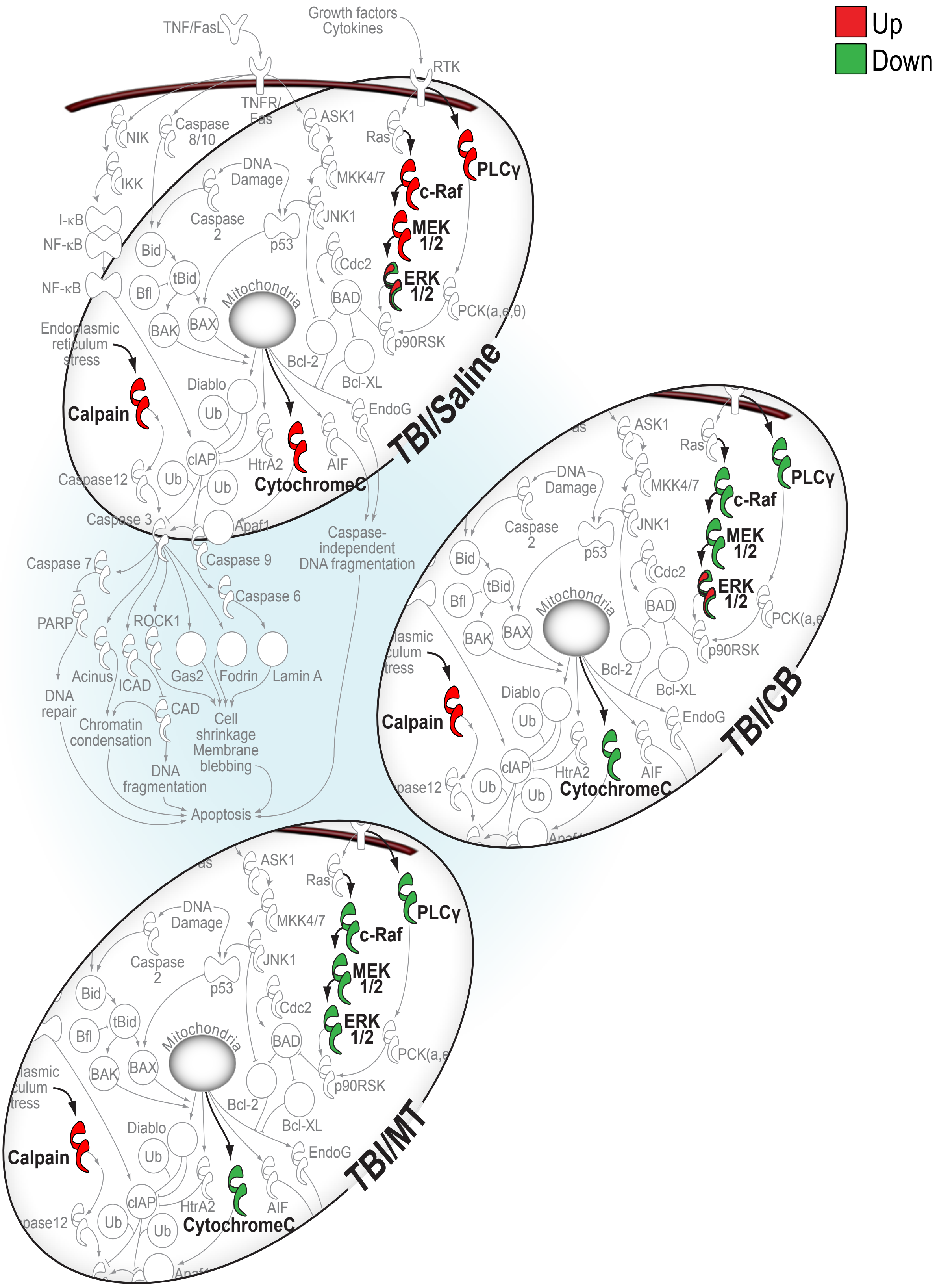

PLCγ [S19]	Phospholipase C, gamma
c-Raf [S20-S22]	V-raf-leukemia viral oncogene 1
MEK 1/2 [S23,S24]	Mitogen-activated protein kinase kinase 1
Cytochrome C [S25,S26]	Cytochrome C oxidase
