# Supplementary material for: Pathway Analysis Reveals Common Pro-Survival Mechanisms of Metyrapone and Carbenoxolone after Traumatic Brain Injury
Source: PLoS One. 2013 Jan 9;8(1):e53230. doi: 10.1371/journal.pone.0053230 (PMC3541279; doi:10.1371/journal.pone.0053230)
Supplement: Figure S4 — Ingenuity pathway analysis of canonical death receptor signaling pathway at 4 h post-TBI. Both metyrapone and carbenoxolone attenuate expression of FLIP, which is known to protect against ischemic cell death. This suggests that drug treatment obviated the need for a protective response. (See Fig. S15 for symbol key). (PDF) [file pone.0053230.s004.pdf]

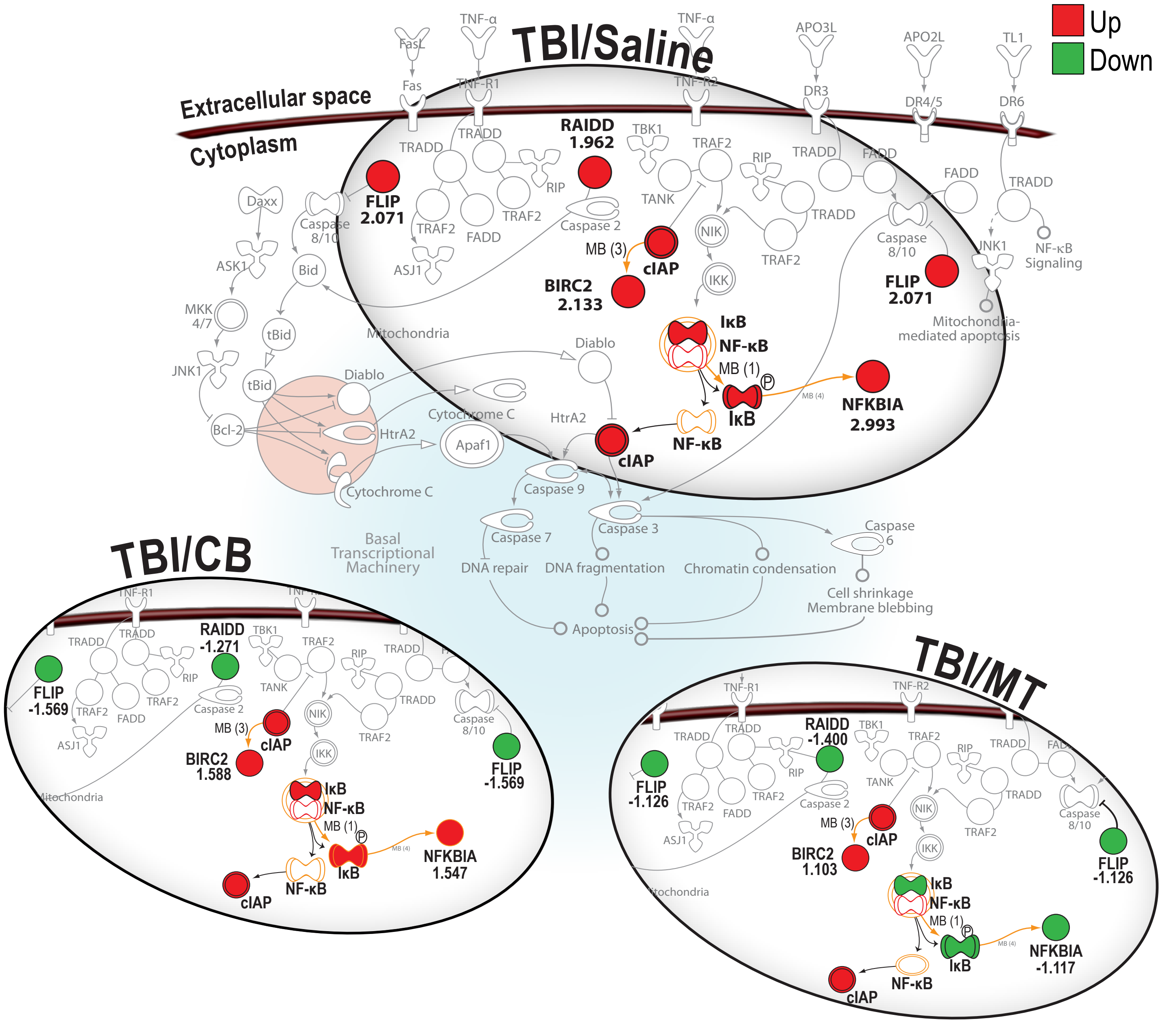


| | |
|---|---|
| FLIP [S27,S28] | Fas-associated death domain-like interleukin-1-β-converting enzyme-inhibitory protein |
| RAIDD [S69] | Receptor-interacting protein (RIP) associated ICH-1/CED-3 homologous protein with a death domain |
