# Supplementary material for: Pathway Analysis Reveals Common Pro-Survival Mechanisms of Metyrapone and Carbenoxolone after Traumatic Brain Injury
Source: PLoS One. 2013 Jan 9;8(1):e53230. doi: 10.1371/journal.pone.0053230 (PMC3541279; doi:10.1371/journal.pone.0053230)
Supplement: Figure S5 — Ingenuity pathway analysis of canonical corticotrophin-releasing hormone (CRH) signaling pathway at 4 h post-TBI. Both metyrapone and carbenoxolone attenuate expression of key cell signaling intermediates. (See Fig. S15 for symbol key). (PDF) [file pone.0053230.s005.pdf]

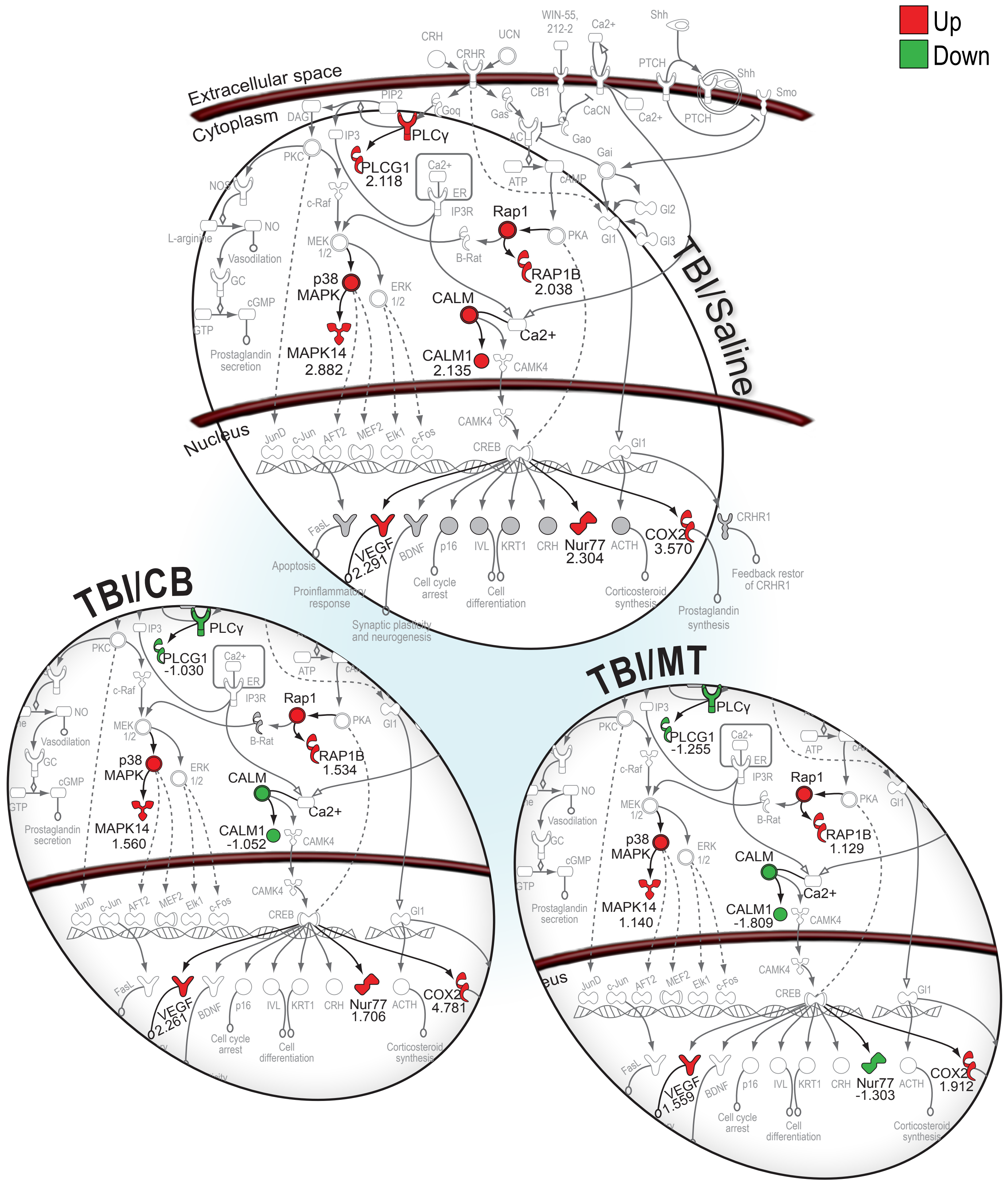

PLCγ [S19]	Phospholipase C gamma
PLCG1 [S29]	Phospholipase C, gamma 1
CALM [S30,S31]	Calmodulin
CALM1 [S32]	Calmodulin 1
