# Supplementary material for: Pathway Analysis Reveals Common Pro-Survival Mechanisms of Metyrapone and Carbenoxolone after Traumatic Brain Injury
Source: PLoS One. 2013 Jan 9;8(1):e53230. doi: 10.1371/journal.pone.0053230 (PMC3541279; doi:10.1371/journal.pone.0053230)
Supplement: Figure S7 — Ingenuity pathway analysis of canonical cyclic AMP response element binding (CREB) protein signaling pathway at 4 h post-TBI. Both metyrapone and carbenoxolone attenuate common genes in this cell survival pathway—a theme that is repeatedly observed in many other injury-induced cell signaling pathways. (See Fig. S15 for symbol key). (PDF) [file pone.0053230.s007.pdf]

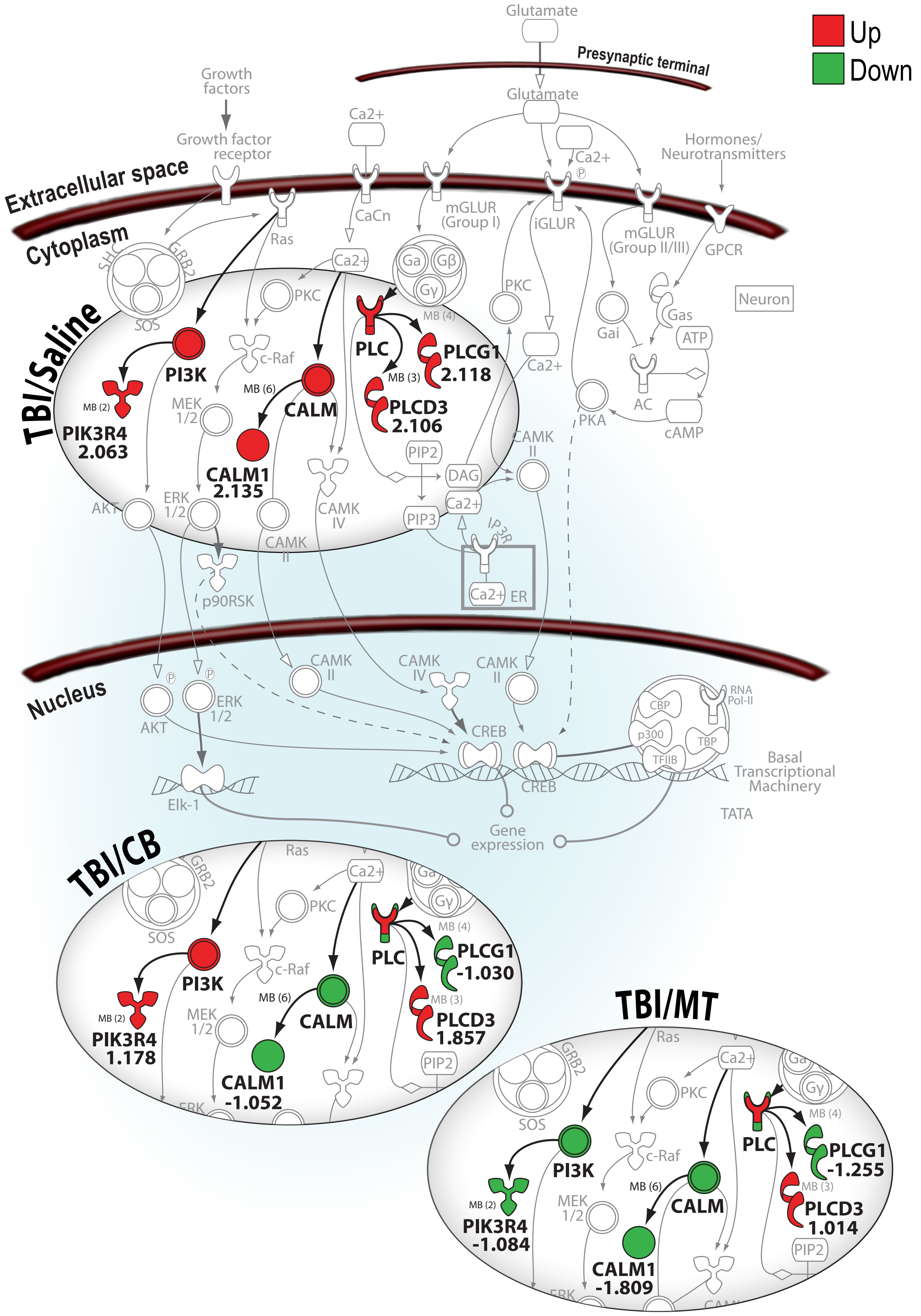

CALM [S30,S31]	Calmodulin
CALM1 [S32]	Calmodulin 1
PLC [S36,S37]	Phospholipase C
PLCG1 [S29]	Phospholipase C, gamma 1
