# Supplementary material for: Pathway Analysis Reveals Common Pro-Survival Mechanisms of Metyrapone and Carbenoxolone after Traumatic Brain Injury
Source: PLoS One. 2013 Jan 9;8(1):e53230. doi: 10.1371/journal.pone.0053230 (PMC3541279; doi:10.1371/journal.pone.0053230)
Supplement: Figure S10 — Ingenuity pathway analysis of canonical phosphotidylinositide 3 kinase/AKT (PI3K/AKT) signaling pathway with 2-fold cut off (A) This illustrates that both metyrapone and carbenoxolone attenuate expression of injury-induced genes in cell survival pathways at 4 h post-TBI. (B) A key cell survival associated gene, NFκB1A is upregulated by both metyrapone and carbenoxolone at 24 h post-TBI compared with TBI alone. (See Fig. S15 for symbol key). (PDF) [file pone.0053230.s010.pdf]

A

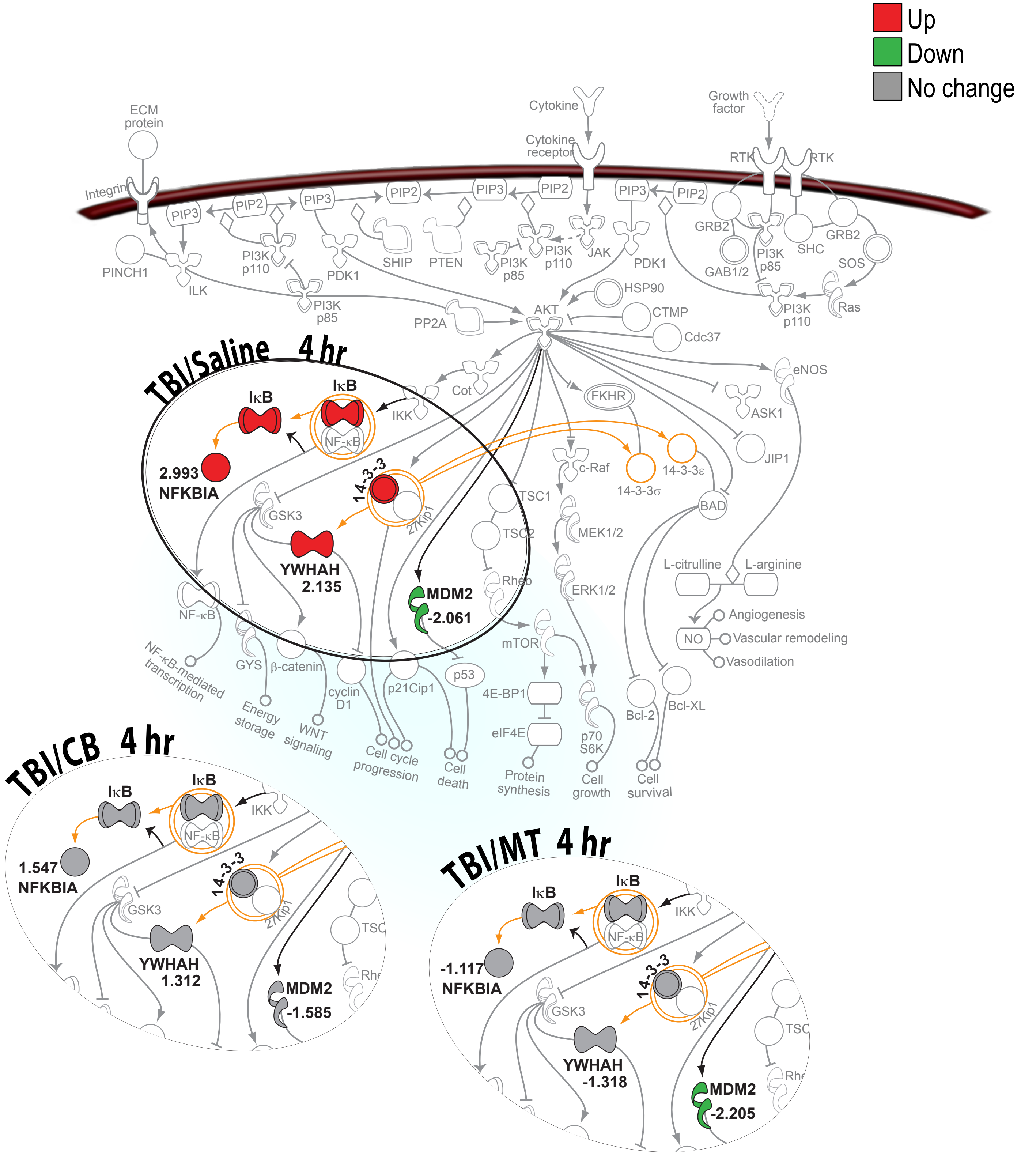

NFKBIA S41,S42	Nuclear factor of kappa light polypeptide gene enhancer in B-cells inhibitor, alpha
IκB S43,S44	Nuclear factor of kappa light polypeptide gene enhancer in B-cells inhibitor, beta
YWHAH S45,S46	Tyrosine 3-monooxygenase/tryptophan 5-monooxygenase activation protein, eta polypeptide
14-3-3 S22,S47-S50	

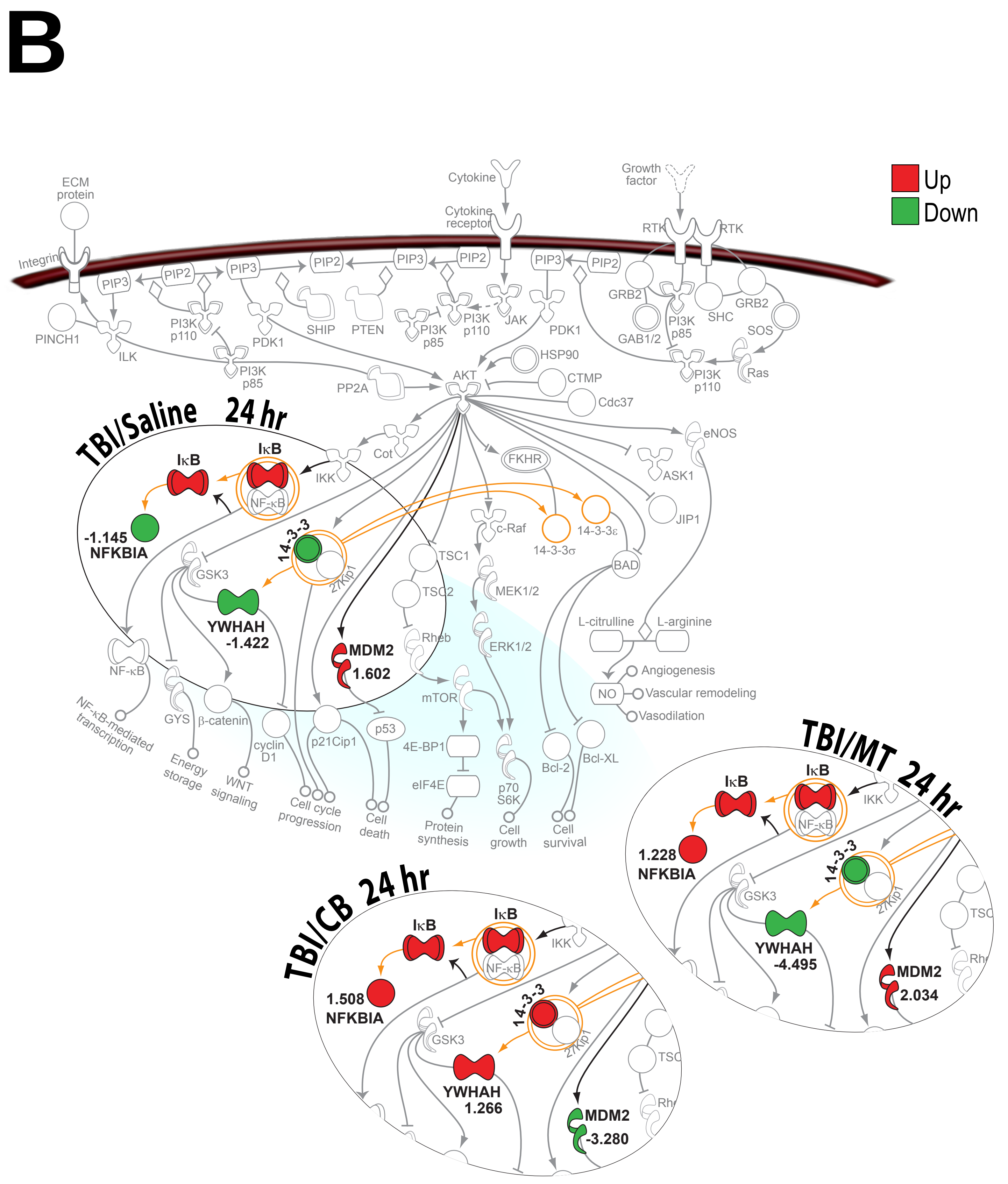

NFKBIA S41,S42	Nuclear factor of kappa light polypeptide gene enhancer in B-cells inhibitor, alpha
IκB S43,S44	Nuclear factor of kappa light polypeptide gene enhancer in B-cells inhibitor, beta
