# Supplementary material for: Pathway Analysis Reveals Common Pro-Survival Mechanisms of Metyrapone and Carbenoxolone after Traumatic Brain Injury
Source: PLoS One. 2013 Jan 9;8(1):e53230. doi: 10.1371/journal.pone.0053230 (PMC3541279; doi:10.1371/journal.pone.0053230)
Supplement: Figure S11 — Ingenuity pathway analysis of axon guidance pathway at 4 h post-TBI. Metyrapone and carbenoxolone attenuate expression of common genes associated with cell survival and regeneration in this pathway. (See Fig. S15 for symbol key). (PDF) [file pone.0053230.s011.pdf]

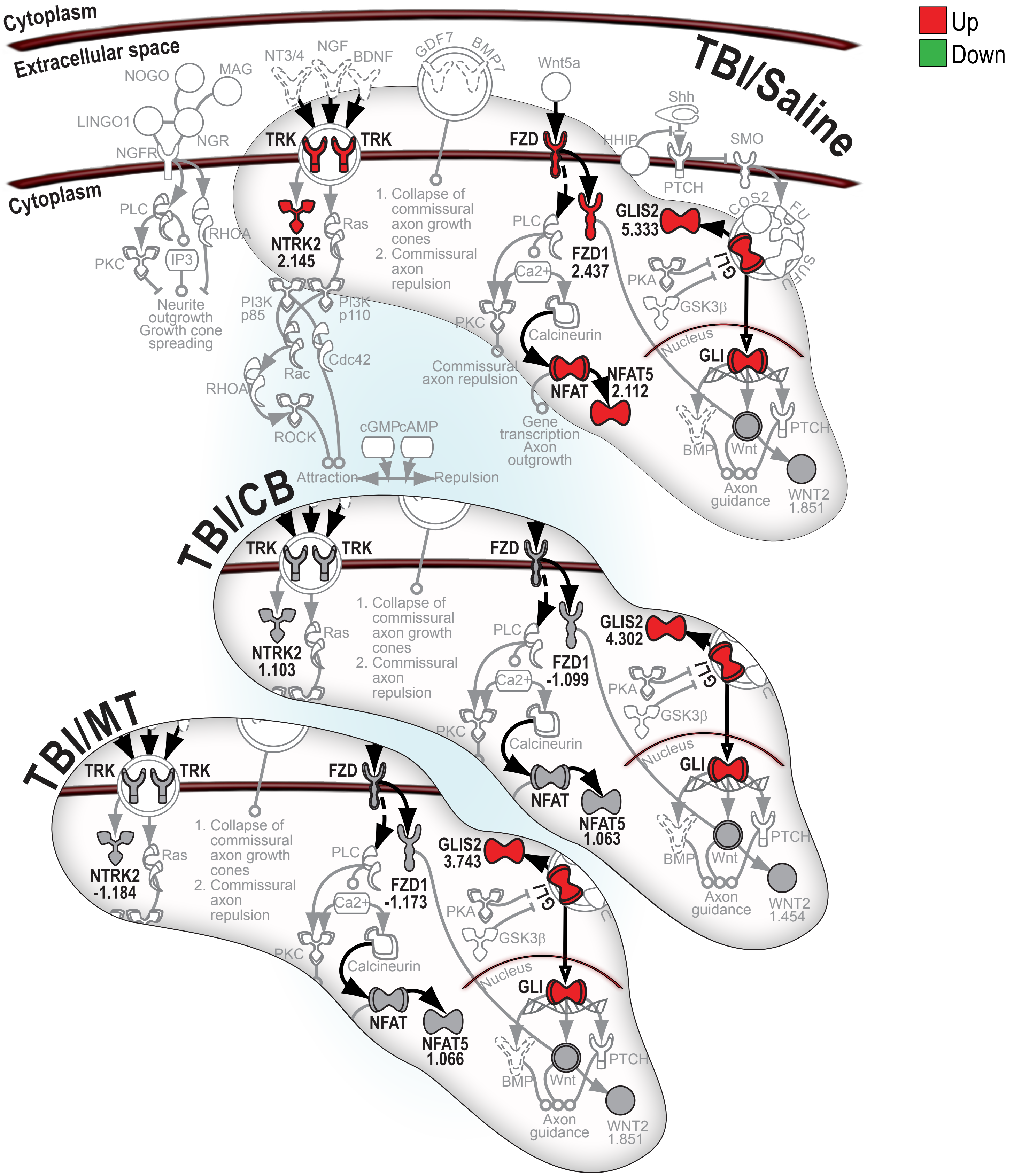


| | |
|---|---|
| NFAT5 [S38] | Nuclear factor of activated T-cells 5 |
| NFAT [S39,S40] | Nuclear factor of activated T-cells |
