# Supplementary material for: Pathway Analysis Reveals Common Pro-Survival Mechanisms of Metyrapone and Carbenoxolone after Traumatic Brain Injury
Source: PLoS One. 2013 Jan 9;8(1):e53230. doi: 10.1371/journal.pone.0053230 (PMC3541279; doi:10.1371/journal.pone.0053230)
Supplement: Figure S13 — Ingenuity pathway analysis of canonical oxidative phosphorylation pathway at 4 h post-TBI. Genes coding for NADH dehydrogenase, the first enzyme in Complex 1 of the mitochondrial electron transport chain are downregulated by both metyrapone and carbenoxolone. This is the site of production of most of the reactive oxygen species in the inner mitochondrial membrane. (See Fig. S15 for symbol key). (PDF) [file pone.0053230.s013.pdf]

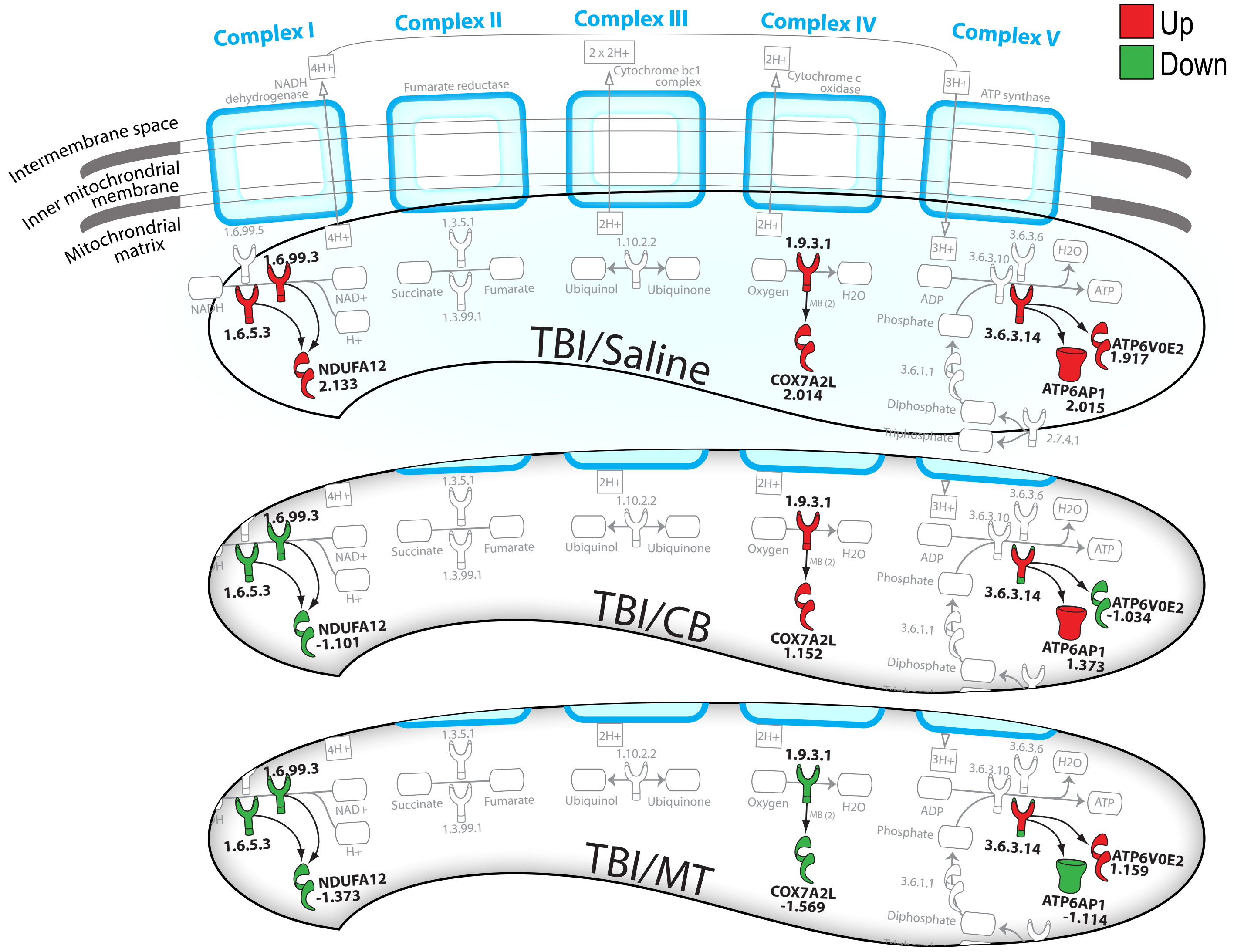

1.6.99.3 [S51]	NADH dehydrogenase, cytochrome C reductase
1.6.5.3 [S52]	NADH dehydrogenase (ubiquinone) Fe-S protein 1, 75kDa (NADH-coenzyme Q reductase)
3.6.3.14 [S53]	H+ transporting two-sector ATPase
NDUFA12 [S54]	NADH dehydrogenase (ubiquinone) 1 alpha subcomplex, 12
