# Supplementary material for: Pathway Analysis Reveals Common Pro-Survival Mechanisms of Metyrapone and Carbenoxolone after Traumatic Brain Injury
Source: PLoS One. 2013 Jan 9;8(1):e53230. doi: 10.1371/journal.pone.0053230 (PMC3541279; doi:10.1371/journal.pone.0053230)
Supplement: Figure S14 — Ingenuity Pathway Analysis of nitric oxide and reactive oxygen species (NO&ROS) signaling pathway in macrophages at 4 h post-TBI with 2-fold cut off. Multiple injury-induced genes in this pathway are downregulated by metyrapone and carbenoxolone treatment compared to TBI alone. (See Fig. S15 for symbol key). (PDF) [file pone.0053230.s014.pdf]

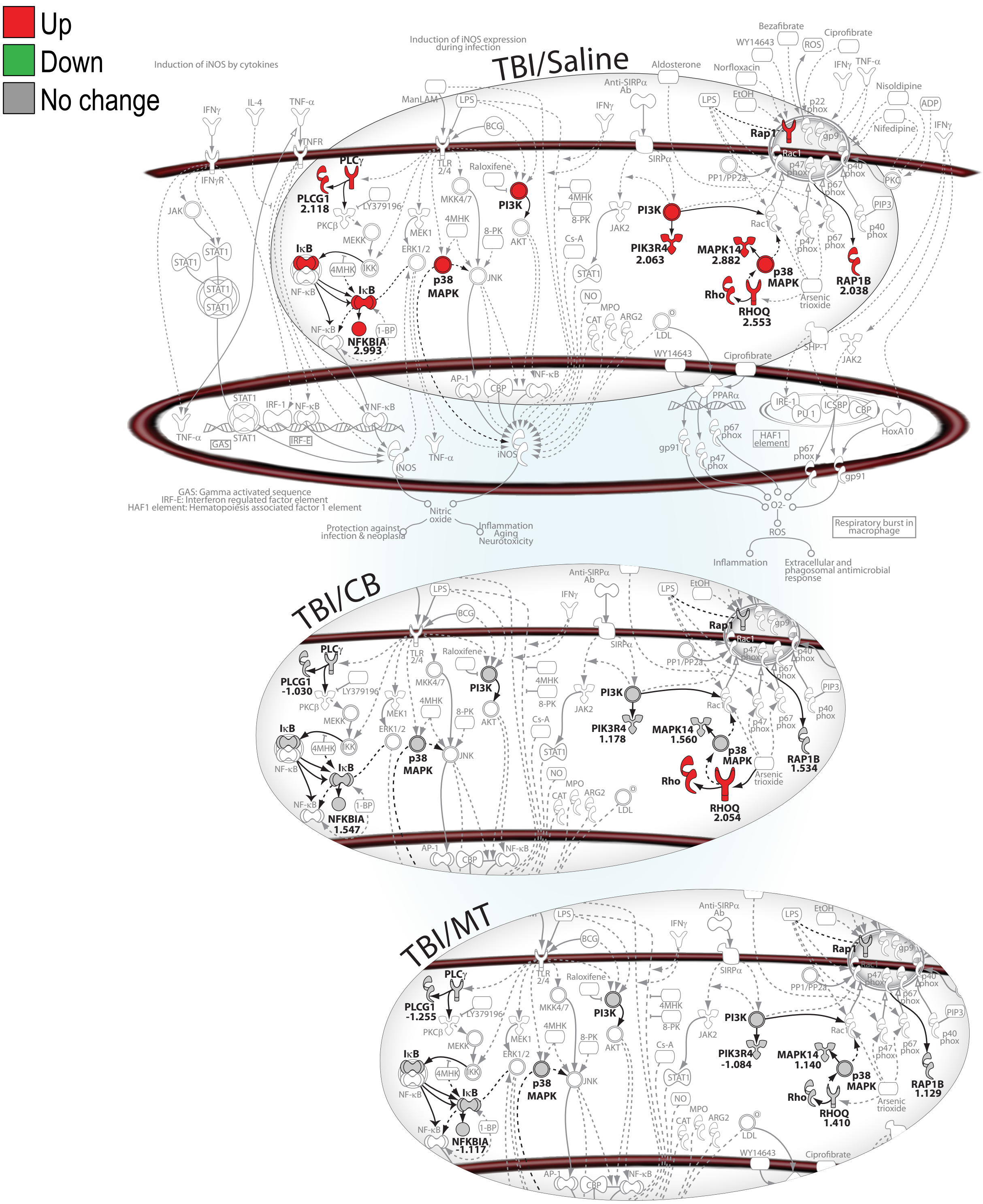

PLCγ [S19]	Phospholipase C gamma
PLCG1 [S29]	Phospholipase C, gamma 1
IκB [S43,S44]	Nuclear factor of kappa light polypeptide gene enhancer in B-cells inhibitor, beta
NFKBIA [S41,S42]	Nuclear factor of kappa light polypeptide gene enhancer in B-cells inhibitor, alpha
MAPK [S42,S51]	Mitogen activated protein kinase
PI3K [S57-S59]	Phosphoinositide-3-kinase, catalytic, alpha polypeptide
PIK3R4 [S60]	Phosphatidylinositol 3 kinase, regulatory subunit, polypeptide 4
Rap1 [S61-S63]	Ras-related protein 1
Rap1b [S64,S65]	GTP-binding protein smg p21B; RAS related protein 1b
MAPK14 [S46,S66-S68]	Mitogen-activated protein kinase 14
