# Supplementary material for: Pathway Analysis Reveals Common Pro-Survival Mechanisms of Metyrapone and Carbenoxolone after Traumatic Brain Injury
Source: PLoS One. 2013 Jan 9;8(1):e53230. doi: 10.1371/journal.pone.0053230 (PMC3541279; doi:10.1371/journal.pone.0053230)

Supporting References

Pathway analysis reveals common pro-survival mechanisms of metyrapone and carbenoxolone after traumatic brain injury

Helen L. Hellmich*

Daniel R. Rojo

Maria-Adelaide Micci

Stacy L. Sell

Deborah R. Boone

Jeanna M. Crookshanks

Douglas S. DeWitt

Brent E. Masel

Donald S. Prough

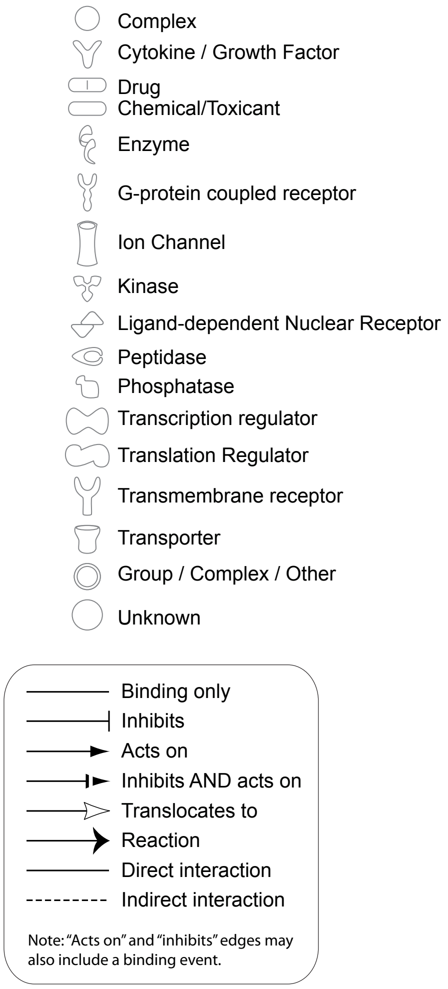

Supplement: Figure S15 — Symbol key for pathways. (PDF) [file pone.0053230.s015.pdf]
