# Supplementary material for: Pathway Analysis Reveals Common Pro-Survival Mechanisms of Metyrapone and Carbenoxolone after Traumatic Brain Injury
Source: PLoS One. 2013 Jan 9;8(1):e53230. doi: 10.1371/journal.pone.0053230 (PMC3541279; doi:10.1371/journal.pone.0053230)
Supplement: Table S1 — Gene Ontology. Genes affected by metyrapone and carbenoxolone treatment participate in diverse cell signaling processes. (PDF) [file pone.0053230.s016.pdf]

Table S1. Gene Ontology. Genes affected by metyrapone and carbenoxolone treatment participate in diverse cell signaling processes

Category		Genes in Category	Genes in List in Category	p-Value*
GO:6629	Lipid metabolism	778	18	0.00000536
GO:44255	Cellular lipid metabolism	652	16	0.00000917
GO:6631	Fatty acid metabolism	208	9	0.0000131
GO:8283	Cell proliferation	956	18	0.0000835
GO:43026	Regulation of caspase activation	16	3	0.000166
GO:43154	Negative regulation of caspase activation	16	3	0.000166
GO:8610	Lipid biosynthesis	299	9	0.000217
GO:7005	Mitochondrion organization and biogenesis	53	4	0.000476
GO:6633	Fatty acid biosynthesis	67	4	0.00116
GO:7006	Mitochondrial membrane organization and biogenesis	31	3	0.00123
GO:42127	Regulation of cell proliferation	635	12	0.00137
GO:6695	Cholesterol biosynthesis	33	3	0.00148
GO:6839	Mitochondrial transport	33	3	0.00148
GO:45941	Positive regulation of transcription	407	9	0.00195
GO:6730	One-carbon compound metabolism	37	3	0.00207
GO:19752	Carboxylic acid metabolism	667	12	0.00207
GO:6082	Organic acid metabolism	669	12	0.00212
GO:16126	Sterol biosynthesis	38	3	0.00224
GO:7050	Cell cycle arrest	81	4	0.00234
GO:16053	Organic acid biosynthesis	83	4	0.00255
GO:46394	Carboxylic acid biosynthesis	83	4	0.00255
GO:45935	Positive regulation of nucleobase, nucleoside, nucleotide and nucleic acid metabolism	424	9	0.00257
GO:50875	Cellular physiological process	12589	97	0.00274
GO:51234	Establishment of localization	3892	40	0.00287

* $p<0.05$
