# Supplementary material for: Pathway Analysis Reveals Common Pro-Survival Mechanisms of Metyrapone and Carbenoxolone after Traumatic Brain Injury
Source: PLoS One. 2013 Jan 9;8(1):e53230. doi: 10.1371/journal.pone.0053230 (PMC3541279; doi:10.1371/journal.pone.0053230)
Supplement: Table S2 — Pathways with Highest # Gene Overlap (partial list). (PDF) [file pone.0053230.s017.pdf]

Table S2. Pathways with Highest # Gene Overlap (partial list)

Pathway Name	#Molecules	Molecule(s)
Role of Macrophages, Fibroblasts and Endothelial Cells in Rheumatoid Arthritis	13	CALM1, CEBPD, FZD1, IL6R, MAPK14, NFAT5, NFKBIA, PIK3R4, PLCD3, PLCG1, PRSS35, VEGFA…
Protein Ubiquitination Pathway	12	BIRC2,NEDD4L, PSMA5, PSMD9, UBA1, UBE2G2, UBE2L3, USP3, USP20, USP31, USP45, VHL
Molecular Mechanisms of Cancer	9	BIRC2, CFLAR, FZD1, HIF1A, MAPK14, NFKBIA, PIK3R4, RAP1B, RHOQ
Axonal Guidance Signaling	9	FZD1, GLIS2, L1CAM, NFAT5, NTRK2, PIK3R4, RAP1B, VEGFA, WNT2
Protein Kinase A Signaling	9	AKAP4, AKAP10, CALM1, NFAT5, NFKBIA, PLCD3, PLCG1, RAP1B, YWHAH
Glycerophospholipid Metabolism	8	AGPAT4, AGPAT5, DGKE, PLA2G3, PLCD3, PLCG1, SPHK2, TAZ
Colorectal Cancer Metastasis Signaling	8	FZDR1, IL6R, MMP15, PIK3R4, PTGS2, RHOQ, VEGFA, WNT2
Cardiac Hypertrophy Signaling	8	ATF6, CALM1, IL6R, MAPK14, PIK3R4, PLCD3, PLCG1, RHOQ
Role of Osteoblasts, Osteoclasts and Chondro-cytes in Rheumatoid Arthritis	8	BIRC2, CALM1, FZD1, MAPK14, NFAT5, NFKBIA, PIK3R4, WNT2
Glucocorticoid Receptor Signaling	7	GTF2H4, MAPK14, NFAT5, NFKBIA, NR3C2, PIK3R4, YWHAH
PPARα/RXRα Activation	7	ACADL, ACOX1, ACVR2B, MAPK14, NFKBIA, PLCD3, PLCG1
Corticotrophin Releasing Hormone Signaling	7	CALM1, MAPK14, NR4A1, PLCG1, PTGS2, RAP1B, VEGFA
Calcium Signaling	7	CALM1, NFAT5, RAP1B, RCAN1, TPM3, TRPC1, TRPC6
Fatty Acid Metabolism	7	ACADL, ACOX1, ALDH3A1, ALDH3A2, CYP4X1, CYP51A1, IWS1
PI3K Signaling in B Lymphocytes	7	ATF6, CALM1, IL4R, NFAT5, NFKBIA, PLCD3, PLCG1
Role of NFAT in Cardiac Hypertrophy	6	CALM1, MAP14, PIK3R4, PLCD3, PLCG1, RCAN1
Glioblastoma Multiforme Signaling	6	FZD1, PIK3R4, PLCD3, PLCG1, RHOQ, WNT2
RANK Signaling in Osteoclasts	6	BIRC2, CALM1, MAPK14, MITF, NFKBIA, PIK3R4
Hypoxia Signaling in the Cardiovascular System	6	HIF1A, NFKBIA, UBE2G2, UBE2L3, VEGFA, VHL
B Cell Receptor Signaling	6	CALM1, EGR1, MAPK14, NFAT5, NFKBIA, PIK3R4
Aldosterone Siglaling in Epithelial Cells	6	ACCN1, ACCN2, NR3C2, PIK3R4, PLCD3, PLCG1
Role of NFAT in Regulation of the Immune Re-sponse	6	CALM1, NFAT5, NFKBIA, PIK3R4, PLCG1, RCAN1
Production of Nitric Oxide and Reactive Oxygen Species in Macrophages	6	MAPK14, NFKBIA, PIK3R4, PLCG1, RAP1B, RHOQ
Purine Metabolism	6	CILP2, DLG3, ENTPD5, NME1, PNPT1, POLD1
Glycerolipid Metabolism	6	AGPAT4, AGPATA5, ALDH3A1, ALDH3A2, DGKE, SPHK2
Phospholipase C Signaling	6	CALM1, NFAT5, PLA2G3, PLCG1, RAP1B, RHOQ
Endothelin-1 Signaling	6	MAPK14, PIK3R4, PLA2G3, PLCD3, PLCG1, PTGS2
HIF1α Signaling	6	HIF1A, MAPK14, MMP15, PIK3R4, VEGFA, VHL
CTLA4 Signaling in Cytotoxic T Lymphocytes	5	AP1G1, CLTA, CLTC, PIK3R4, PLCG1
Valine, Leucine and Isoleucine Degradation	5	ACADL, ALDH3A1, ALDH3A2, IWS1, OXCT1
CD28 Signaling in T Helper Cells	5	CALM1, NFAT5, NFKBIA, PIK3R4, PLCG1, RCAN1
Tryptophan Metabolism	5	ALDH3A1, ALDH3A2, CYP4X1, CYP51A1, PTGS2
Arachidonic Acid Metabolism	5	CBR3, CYP4X1, CYP51A12, PLA2G3, PTGS2
IL-10 Signaling	5	IL10RA, IL4R, MAPK14, NFKBIA, SP1
