# Supplementary material for: Pathway Analysis Reveals Common Pro-Survival Mechanisms of Metyrapone and Carbenoxolone after Traumatic Brain Injury
Source: PLoS One. 2013 Jan 9;8(1):e53230. doi: 10.1371/journal.pone.0053230 (PMC3541279; doi:10.1371/journal.pone.0053230)
Supplement: Table S3 — Top significant pathways of interest with biological relevance. (PDF) [file pone.0053230.s018.pdf]

Table S3. Top significant pathways of interest with biological relevance

Pathways/Functions of Interest	Top Pathways With Biological Relevance
Cell Survival Pathways	Protein Ubiquitination
Apoptosis Signaling	Hypoxia Signaling in the Cardiovascular System
Death Receptor Signaling	TWEAK Signaling
Amyloid Receptor Signaling	Corticotrophin Releasing Hormone Signaling
Inflammation	Death Receptor Signaling
Oxidative Stress	Role of Macrophages, Fibroblasts and Endothelial Cells in Rheumatoid Arthritis
Synaptic Plasticity	Axonal Guidance Signaling
Development	Apoptosis Signaling
Neurogenesis	Glycerophospholipid Metabolism
	IL-10 Signaling
	TNFR1 Signaling
	Protein Kinase A Signaling

Pathways associated with cell survival or death are identified through IPA
