# Supplementary material for: Pathway Analysis Reveals Common Pro-Survival Mechanisms of Metyrapone and Carbenoxolone after Traumatic Brain Injury
Source: PLoS One. 2013 Jan 9;8(1):e53230. doi: 10.1371/journal.pone.0053230 (PMC3541279; doi:10.1371/journal.pone.0053230)
Supplement: References S1 — Supplementary References (PDF) [file pone.0053230.s019.pdf]

Supporting References

S1. Zheng,J. et al. Clathrin-dependent endocytosis is required for TrkB-dependent Akt-mediated neuronal protection and dendritic growth. J Biol. Chem. 283, 13280-13288 (2008).

S2. Liu,B. et al. Ischemic insults direct glutamate receptor subunit 2-lacking AMPA receptors to synaptic sites. J Neurosci. 26, 5309-5319 (2006).

S3. So,C.W., Lin,M., Ayton,P.M., Chen,E.H., & Cleary,M.L. Dimerization contributes to oncogenic activation of MLL chimeras in acute leukemias. Cancer Cell 4, 99-110 (2003).

S4. Fallon,L. et al. A regulated interaction with the UIM protein Eps15 implicates parkin in EGF receptor trafficking and PI(3)K-Akt signalling. Nat Cell Biol. 8, 834-842 (2006).

S5. Muller,A.J. et al. Targeted disruption of the murine Bin1/Amphiphysin II gene does not disable endocytosis but results in embryonic cardiomyopathy with aberrant myofibril formation. Mol. Cell Biol. 23, 4295-4306 (2003).

S6. Monstad,S.E., Knudsen,A., Salvesen,H.B., Aarseth,J.H., & Vedeler,C.A. Onconeural antibodies in sera from patients with various types of tumours. Cancer Immunol. Immunother. 58, 1795-1800 (2009).

S7. Zupanc,G.K. & Clint,S.C. Radial glia-mediated up-regulation of somatostatin in the regenerating adult fish brain. Neurosci. Lett. 309, 149-152 (2001).

S8. Braun,H., Schulz,S., & Hollt,V. Expression changes of somatostatin receptor subtypes sst2A, sst2B, sst3 and sst4 after a cortical contusion trauma in rats. Brain Res. 930, 191-199 (2002).

S9. Holmberg,K.H. & Patterson,P.H. Leukemia inhibitory factor is a key regulator of astrocytic, microglial and neuronal responses in a low-dose pilocarpine injury model. Brain Res. 1075, 26-35 (2006).

S10. Duszczyk,M. et al. Changes in the NPY immunoreactivity in gerbil hippocampus after hypoxic and ischemic preconditioning. Neuropeptides 43, 31-39 (2009).

S11. Zhong,S., Machida,K., Tsukamoto,H., & Johnson,D.L. Alcohol induces RNA polymerase III-dependent transcription through c-Jun by co-regulating TATA-binding protein (TBP) and Brf1 expression. J Biol. Chem. 286, 2393-2401 (2011).

S12. Byun,H.O. et al. Cathepsin D and eukaryotic translation elongation factor 1 as promising markers of cellular senescence. Cancer Res. 69, 4638-4647 (2009).

S13. Kusumawidjaja,G. et al. Basic transcription factor 3 (BTF3) regulates transcription of tumor-associated genes in pancreatic cancer cells. Cancer Biol. Ther 6, 367-376 (2007).

S14. Brockstedt,E., Otto,A., Rickers,A., Bommert,K., & Wittmann-Liebold,B. Preparative high-resolution two-dimensional electrophoresis enables the identification of RNA polymerase B transcription factor 3 as an apoptosis-associated protein in the human BL60-2 Burkitt lymphoma cell line. J Protein Chem. 18, 225-231 (1999).

S15. Hu,X. et al. Peroxiredoxin-2 protects against 6-hydroxydopamine-induced dopaminergic neurodegeneration via attenuation of the apoptosis signal-regulating kinase (ASK1) signaling cascade. J Neurosci. 31, 247-261 (2011).

S16. Hattori,F., Murayama,N., Noshita,T., & Oikawa,S. Mitochondrial peroxiredoxin-3 protects hippocampal neurons from excitotoxic injury in vivo. J Neurochem. 86, 860-868 (2003).

S17. Jahani-Asl,A. et al. The mitochondrial inner membrane GTPase, optic atrophy 1 (Opa1), restores mitochondrial morphology and promotes neuronal survival following excitotoxicity. J Biol. Chem. 286, 4772-4782 (2011).

S18. Papakonstantinou,T. et al. Expression of protein tyrosine phosphatase-like molecule ICA512/IA-2 induces growth arrest in yeast cells and transfected mammalian cell lines. J Autoimmun. 17, 51-61 (2001).

S19. Carman,H.M., Dhillon,H.S., Zhang,D., Geddes,J.W., & Prasad,R.M. Regional levels of phospholipase Cgamma after fluid percussion brain injury in the rat. Brain Res. 808, 116-119 (1998).

S20. Planchamp,V. et al. BAG1 promotes axonal outgrowth and regeneration in vivo via Raf-1 and reduction of ROCK activity. Brain 131, 2606-2619 (2008).

S21. Koh,P.O. Estradiol prevents the injury-induced decrease of 90 ribosomal S6 kinase (p90RSK) and Bad phosphorylation. Neurosci. Lett. 412, 68-72 (2007).

S22. Koh,P.O. Melatonin attenuates the cerebral ischemic injury via the MEK/ERK/p90RSK/bad signaling cascade. J Vet. Med. Sci. 70, 1219-1223 (2008).

S23. Wang,X. et al. Significant neuroprotection against ischemic brain injury by inhibition of the MEK1 protein kinase in mice: exploration of potential mechanism associated with apoptosis. J Pharmacol Exp Ther 304, 172-178 (2003).

S24. Wang,Z.Q., Wu,D.C., Huang,F.P., & Yang,G.Y. Inhibition of MEK/ERK 1/2 pathway reduces pro-inflammatory cytokine interleukin-1 expression in focal cerebral ischemia. Brain Res. 996, 55-66 (2004).

S25. Wagner,A.K. et al. CSF Bcl-2 and cytochrome C temporal profiles in outcome prediction for adults with severe TBI. J Cereb. Blood Flow Metab(2011).

S26. Shojo,H. et al. Genetic and histologic evidence implicates role of inflammation in traumatic brain injury-induced apoptosis in the rat cerebral cortex following moderate fluid percussion injury. Neuroscience 171, 1273-1282 (2010).

S27. Northington,F.J. et al. Necrostatin decreases oxidative damage, inflammation, and injury after neonatal HI. J Cereb. Blood Flow Metab 31, 178-189 (2011).

S28. Taoufik,E. et al. FLIP(L) protects neurons against in vivo ischemia and in vitro glucose deprivation-induced cell death. J Neurosci. 27, 6633-6646 (2007).

S29. Cortese,G.P., Barrientos,R.M., Maier,S.F., & Patterson,S.L. Aging and a peripheral immune challenge interact to reduce mature brain-derived neurotrophic factor and activation of TrkB, PLCgamma1, and ERK in hippocampal synaptoneurosomes. J Neurosci. 31, 4274-4279 (2011).

S30. Xu,J., Zhang,Q.G., Li,C., & Zhang,G.Y. Subtoxic N-methyl-D-aspartate delayed neuronal death in ischemic brain injury through TrkB receptor- and calmodulin-mediated PI-3K/Akt pathway activation. Hippocampus 17, 525-537 (2007).

S31. Dai,C. et al. Functional identification of neuroprotective molecules. PLoS. One. 5, e15008 (2010).

S32. Cifuentes,E. et al. Physical and functional interaction of androgen receptor with calmodulin in prostate cancer cells. Proc Natl. Acad. Sci. U. S. A 101, 464-469 (2004).

S33. Atkins,C.M. et al. Modulation of the cAMP signaling pathway after traumatic brain injury. Exp Neurol. 208, 145-158 (2007).

S34. Wang,S.M. & Yang,W.L. Circulating hormone adrenomedullin and its binding protein protect neural cells from hypoxia-induced apoptosis. Biochim. Biophys. Acta 1790, 361-367 (2009).

S35. McCullough,L. et al. Neuroprotective function of the PGE2 EP2 receptor in cerebral ischemia. J Neurosci. 24, 257-268 (2004).

S36. Sieber,F.E., Traystman,R.J., & Martin,L.J. Delayed neuronal death after global incomplete ischemia in dogs is accompanied by changes in phospholipase C protein expression. J. Cereb. Blood Flow Metab. 17, 527-533 (1997).

S37. Rogido,M. et al. Fructose-1,6-biphosphate prevents excitotoxic neuronal cell death in the neonatal mouse brain. Brain Res. Dev. Brain Res. 140, 287-297 (2003).

S38. Shaterian,A., Borboa,A., Coimbra,R., Baird,A., & Eliceiri,B.P. Non-invasive detection of spatio-temporal activation of SBE and NFAT5 promoters in transgenic reporter mice following stroke. Neuropathology. (2011).

S39. Vashishta,A. et al. Nuclear factor of activated T-cells isoform c4 (NFATc4/NFAT3) as a mediator of antiapoptotic transcription in NMDA receptor-stimulated cortical neurons. J Neurosci. 29, 15331-15340 (2009).

S40. Fernandez,A.M., Fernandez,S., Carrero,P., Garcia-Garcia,M., & Torres-Aleman,I. Calcineurin in reactive astrocytes plays a key role in the interplay between proinflammatory and anti-inflammatory signals. J Neurosci. 27, 8745-8756 (2007).

S41. Shen,W.H., Zhang,C.Y., & Zhang,G.Y. Antioxidants attenuate reperfusion injury after global brain ischemia through inhibiting nuclear factor-kappa B activity in rats. Acta Pharmacol Sin. 24, 1125-1130 (2003).

S42. Sanz,O., Acarin,L., Gonzalez,B., & Castellano,B. NF-kappaB and IkappaBalpha expression following traumatic brain injury to the immature rat brain. J Neurosci. Res. 67, 772-780 (2002).

S43. Hake,I. et al. Neuroprotection and enhanced neurogenesis by extract from the tropical plant Knema laurina after inflammatory damage in living brain tissue. J Neuroimmunol. 206, 91-99 (2009).

S44. Greco,R. et al. IkappaB-alpha expression following transient focal cerebral ischemia is modulated by nitric oxide. Brain Res. 1372, 145-151 (2011).

S45. Daigeler,A. et al. Heterogeneous in vitro effects of doxorubicin on gene expression in primary human liposarcoma cultures. BMC. Cancer 8, 313 (2008).

S46. Oyeniran,C. & Tanfin,Z. MAPK14 cooperates with MAPK3/1 to regulate endothelin-1-mediated prostaglandin synthase 2 induction and survival in leiomyoma but not in normal myometrial cells. Biol. Reprod. 84, 495-504 (2011).

S47. Koh,P.O. Melatonin attenuates the focal cerebral ischemic injury by inhibiting the dissociation of pBad from 14-3-3. J Pineal Res. 44, 101-106 (2008).

S48. Chan,P.H. Mitochondria and neuronal death/survival signaling pathways in cerebral ischemia. Neurochem. Res. 29, 1943-1949 (2004).

S49. Morrison,D.K. The 14-3-3 proteins: integrators of diverse signaling cues that impact cell fate and cancer development. Trends Cell Biol. 19, 16-23 (2009).

S50. Chen,J., Lee,C.T., Errico,S.L., Becker,K.G., & Freed,W.J. Increases in expression of 14-3-3 eta and 14-3-3 zeta transcripts during neuroprotection induced by delta9-tetrahydrocannabinol in AF5 cells. J Neurosci. Res. 85, 1724-1733 (2007).

S51. Kaniuga,Z. THE TRANSFORMATION OF MITOCHONDRIAL NADH DEHYDROGENASE INTO NADH: CYTOCHROME C OXIDOREDUCTASE. Biochim. Biophys. Acta 73, 550-564 (1963).

S52. Ono,H., Nakamura,H., & Matsuzaki,M. A NADH dehydrogenase ubiquinone flavoprotein is decreased in patients with dilated cardiomyopathy. Intern. Med. 49, 2039-2042 (2010).

S53. Blair,A., Ngo,L., Park,J., Paulsen,I.T., & Saier,M.H., Jr. Phylogenetic analyses of the homologous transmembrane channel-forming proteins of the F0F1-ATPases of bacteria, chloroplasts and mitochondria. Microbiology 142 (Pt 1), 17-32 (1996).

S54. Dieteren,C.E. et al. Subunits of mitochondrial complex I exist as part of matrix- and membrane-associated subcomplexes in living cells. J Biol. Chem. 283, 34753-34761 (2008).

S55. Patel,N.J., Chen,M.J., & Russo-Neustadt,A.A. Norepinephrine and nitric oxide promote cell survival signaling in hippocampal neurons. Eur. J Pharmacol 633, 1-9 (2010).

S56. Tomaselli,B., Podhraski,V., Heftberger,V., Bock,G., & Baier-Bitterlich,G. Purine nucleoside-mediated protection of chemical hypoxia-induced neuronal injuries involves p42/44 MAPK activation. Neurochem. Int. 46, 513-521 (2005).

S57. Xu,X. et al. Neuroprotective effect of humanin on cerebral ischemia/reperfusion injury is mediated by a PI3K/Akt pathway. Brain Res. 1227, 12-18 (2008).

S58. Taylor,J.M., Ali,U., Iannello,R.C., Hertzog,P., & Crack,P.J. Diminished Akt phosphorylation in neurons lacking glutathione peroxidase-1 (Gpx1) leads to increased susceptibility to oxidative stress-induced cell death. J Neurochem. 92, 283-293 (2005).

S59. Liu,C. et al. Neuroprotection by baicalein in ischemic brain injury involves PTEN/AKT pathway. J

Neurochem. 112, 1500-1512 (2010).

S60. Huang,J. et al. Frequent genetic abnormalities of the PI3K/AKT pathway in primary ovarian cancer predict patient outcome. Genes Chromosomes. Cancer 50, 606-618 (2011).

S61. Bailey,C.L., Kelly,P., & Casey,P.J. Activation of Rap1 promotes prostate cancer metastasis. Cancer Res. 69, 4962-4968 (2009).

S62. Banerjee,R., Henson,B.S., Russo,N., Tsodikov,A., & D'Silva,N.J. Rap1 mediates galanin receptor 2-induced proliferation and survival in squamous cell carcinoma. Cell Signal. 23, 1110-1118 (2011).

S63. Obara,Y., Horgan,A.M., & Stork,P.J. The requirement of Ras and Rap1 for the activation of ERKs by cAMP, PACAP, and KCl in cerebellar granule cells. J Neurochem. 101, 470-482 (2007).

S64. Sun,L. et al. Rap1b GTPase ameliorates glucose-induced mitochondrial dysfunction. J Am. Soc. Nephrol. 19, 2293-2301 (2008).

S65. Chen,Y. et al. A critical role of Rap1b in B-cell trafficking and marginal zone B-cell development. Blood 111, 4627-4636 (2008).

S66. Matrone,A. et al. p38alpha is required for ovarian cancer cell metabolism and survival. Int. J Gynecol. Cancer 20, 203-211 (2010).

S67. Porras,A. et al. P38 alpha mitogen-activated protein kinase sensitizes cells to apoptosis induced by different stimuli. Mol. Biol. Cell 15, 922-933 (2004).

S68. Chapuy,B. et al. Multikinase inhibitor sorafenib exerts cytocidal efficacy against Non-Hodgkin lymphomas associated with inhibition of MAPK14 and AKT phosphorylation. Br. J Haematol. 152, 401-412 (2011).

S69. Berube,C. et al. Apoptosis caused by p53-induced protein with death domain (PIDD) depends on the death adapter protein RAIDD. PNAS 102, 14314-14320 (2005)
