# Supplementary material for: Pathway Analysis Reveals Common Pro-Survival Mechanisms of Metyrapone and Carbenoxolone after Traumatic Brain Injury
Source: PLoS One. 2013 Jan 9;8(1):e53230. doi: 10.1371/journal.pone.0053230 (PMC3541279; doi:10.1371/journal.pone.0053230)
Supplement: Figure S8 — Ingenuity pathway analysis of NFAT in regulation of immune response pathway at 24 h post-TBI. Similar to the PI3K/AKT pathway (also see Figure S10b), cell survival-associated genes downregulated after TBI alone are upregulated by metyrapone or carbenoxolone treatment 24 h after injury. (See Fig. S15 for symbol key). (PDF) [file pone.0053230.s008.pdf]

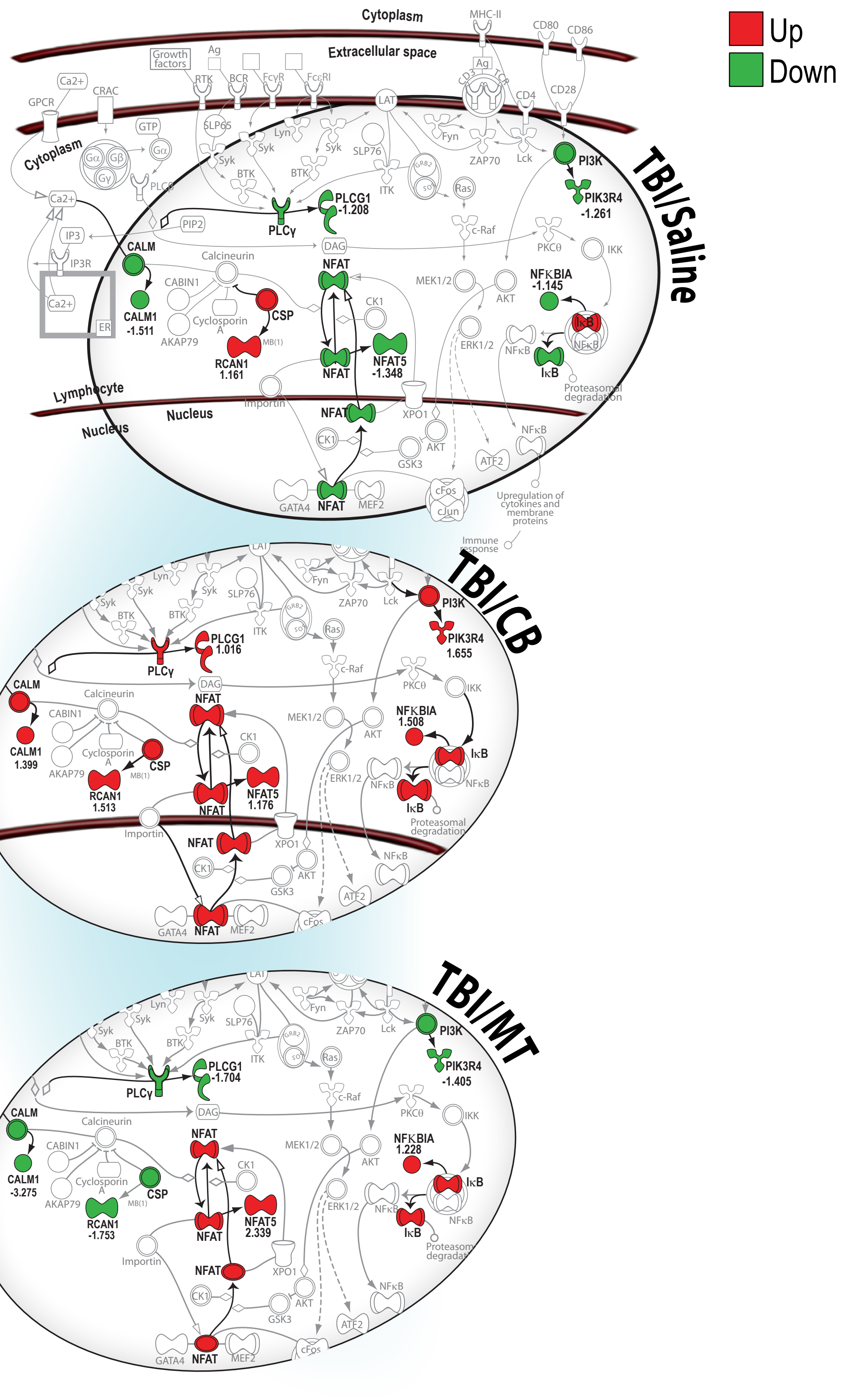

NFAT5 [S38]	Nuclear factor of activated T-cells 5
NFAT [S39,S40]	Nuclear factor of activated T-cells
NFKBIA [S41,S42]	Nuclear factor of kappa light polypeptide gene enhancer in B-cells inhibitor, alpha
IκB [S43,S44]	Nuclear factor of kappa light polypeptide gene enhancer in B-cells inhibitor, beta
